# Dynamic Bayesian Networks for Integrating Multi-omics Time Series Microbiome Data

**DOI:** 10.1128/mSystems.01105-20

**Published:** 2021-03-30

**Authors:** Daniel Ruiz-Perez, Jose Lugo-Martinez, Natalia Bourguignon, Kalai Mathee, Betiana Lerner, Ziv Bar-Joseph, Giri Narasimhan

**Affiliations:** a Florida International University, Bioinformatics Research Group (BioRG), Miami, Florida, USA; b Carnegie Mellon University, Computational Biology Department, School of Computer Science, Pittsburgh, Pennsylvania, USA; c Florida International University, College of Engineering and Computing, Miami, Florida, USA; d National Technological University, Buenos Aires, Argentina; e Florida International University, Herbert Wertheim College of Medicine, Miami, Florida, USA; f Florida International University, Biomolecular Sciences Institute, Miami, Florida, USA; Columbia University Irving Medical Center

**Keywords:** longitudinal microbiome analysis, multi-omic integration, microbial composition prediction, dynamic Bayesian networks, temporal alignment

## Abstract

A key challenge in the analysis of longitudinal microbiome data is the inference of temporal interactions between microbial taxa, their genes, the metabolites that they consume and produce, and host genes. To address these challenges, we developed a computational pipeline, a pipeline for the analysis of longitudinal multi-omics data (PALM), that first aligns multi-omics data and then uses dynamic Bayesian networks (DBNs) to reconstruct a unified model. Our approach overcomes differences in sampling and progression rates, utilizes a biologically inspired multi-omic framework, reduces the large number of entities and parameters in the DBNs, and validates the learned network. Applying PALM to data collected from inflammatory bowel disease patients, we show that it accurately identifies known and novel interactions. Targeted experimental validations further support a number of the predicted novel metabolite-taxon interactions.

**IMPORTANCE** While a number of large consortia collect and profile several different types of microbiome and genomic time series data, very few methods exist for joint modeling of multi-omics data sets. We developed a new computational pipeline, PALM, which uses dynamic Bayesian networks (DBNs) and is designed to integrate multi-omics data from longitudinal microbiome studies. When used to integrate sequence, expression, and metabolomics data from microbiome samples along with host expression data, the resulting models identify interactions between taxa, their genes, and the metabolites that they produce and consume, as well as their impact on host expression. We tested the models both by using them to predict future changes in microbiome levels and by comparing the learned interactions to known interactions in the literature. Finally, we performed experimental validations for a few of the predicted interactions to demonstrate the ability of the method to identify novel relationships and their impact.

## INTRODUCTION

Microbiomes are communities of microbes inhabiting an environmental niche. The study of microbial communities offers a powerful approach for inferring their impact on the host environment and their role in specific diseases and health (50). Metagenomics involves analyzing sequenced reads from the whole metagenome in a microbial community in order to determine a detailed profile of microbial taxa (1). More recently, additional types of biological data are being profiled in microbiome studies, including metatranscriptomics, which involves surveying the complete metatranscriptome of the microbial community (2), metabolomics, which involves profiling the entire set of small molecules (metabolites) present in the microbiome’s environmental niche (3), and host transcriptomics, which provides information about the levels of genes expressed in the host (4).

The goal of the second phase of the Human Microbiome Project (HMP) (5), called the integrative Human Microbiome Project (iHMP) (6), is to generate longitudinal multi-omics data sets as a means to study the dynamics of the microbiome and the host across select diseases, including preterm births, type 2 diabetes, and irritable bowel disorders.

A major challenge in microbiome data analysis is the integration of multi-omics data sets (7, 51). Most multi-omic studies focus on a separate analysis of each omics data set without building a unified model (8). There have been some attempts (9–13) and tools (14, 15) to facilitate the analysis, but there is still much room for improvement regarding reproducibility, flexibility, and biological validity (7, 16, 17).

Deep learning approaches for integrating multi-omics (18) have also been developed, but their lack of interpretability prevents these models from providing insights into the interplay of the different omics entities, with the exception of MMvec (19), but it combines only metabolites and taxa. Even partial least-squares models have been used to facilitate this integration (20), but they have their own set of limitations depending on the underlying data generation model and are prone to provide spurious results when applied to high-dimensional data (21).

In addition, microbiomes are inherently dynamic, and so to fully understand the complex interactions that take place within these communities, longitudinal microbiome data appear to be critical (22). Many attempts have been made to analyze data from longitudinal studies (12, 13, 23); however, these approaches do not attempt to study interactions between taxa. An alternative approach involves the use of dynamical systems such as the generalized Lotka-Volterra (gLV) models (24, 25); however, the large set of parameters in these models diminishes their utility for probabilistic inference.

Previously, we have shown that probabilistic graphical models, specifically dynamic Bayesian networks (DBNs), can be used to study metagenomic sequence data from microbiome studies, leading to models that can accurately predict future changes as well as identify interactions within the microbiome (26). However, these prior methods were able to analyze only a single omic data set. Here, we present a new pipeline for the analysis of longitudinal multi-omics data (PALM), which, in addition to modeling metagenomics interactions, can incorporate time series metatranscriptomics, metabolomics, and host expression data to train an integrated model of microbiome-host interactions.

A number of challenges are associated with such large-scale integration. First, modeling such data leads to a considerable increase in the size of the model and the number of parameters in the DBN, which grows as the product of the number of entities in each omics data set grows. More complex models make the computation less tractable and harder to interpret. Additionally, such a large number of nodes and parameters can lead to overfitting. PALM overcomes these challenges by restricting the set of allowable interactions (edges) between the omics entities based on sound biological assumptions and by relying on continuous representation and alignment to integrate a large set of observations when training a specific model.

An additional challenge with modeling microbiomes is the difficulty of validating the model’s predictions. To address this, PALM uses *in silico* approaches employing multiple public databases (a genomic sequence database and a metabolic pathway database) and recently proposed software tools for the validation.

Applying PALM to inflammatory bowel disease (IBD) data led to models that correctly predicted microbiome abundance levels and identified known and novel interactions. Statistical validations indicated that PALM can accurately recover known interactions and improves upon prior approaches. We also experimentally validated a few of the highly scoring metabolite-taxon interactions predicted by the model.

## RESULTS

**FIG 1:**
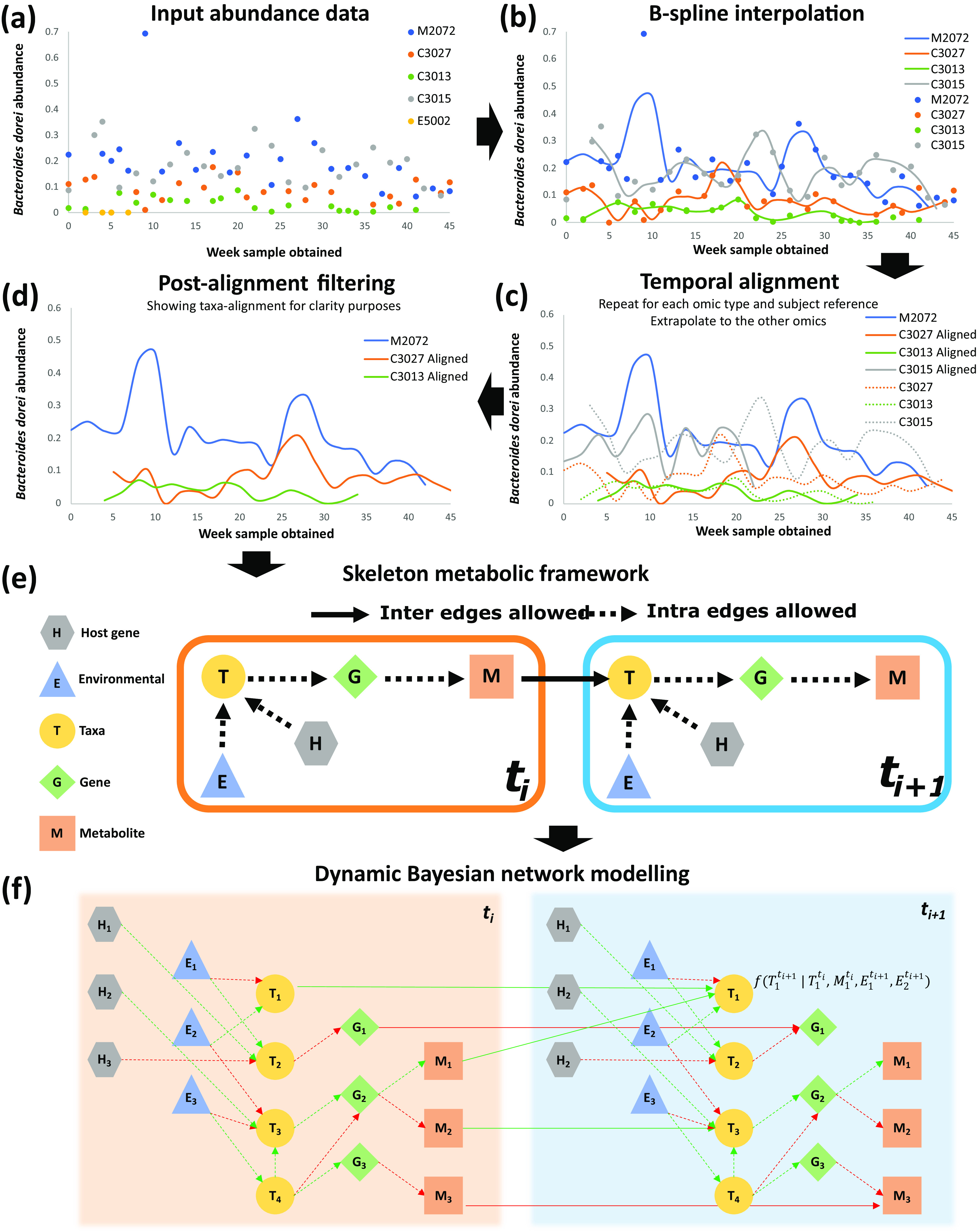
Computational pipeline proposed in this work. For simplicity, the figure shows only the microbial taxon Bacteroides dorei at each step in the pipeline from a set of five individual samples (subjects M2072, C3027, C3013, C3015, and E5002) of the IBD data set. (a) The relative abundance for each sample measured at potentially nonuniform intervals is the input of the pipeline. (b) Cubic B-spline curve for each individual sample. Subject E5002 (yellow) does not contain enough measured time points and was excluded from further analysis. The remaining smoothed curves enable principled estimation of unobserved time points and interpolation at specified intervals. (c) Temporal alignment of all taxa of each individual against the optimal reference subject (subject M2072 in blue). The learned warping function is extrapolated to all other omics data (e.g., genes and metabolites) of each subject. This process is then repeated, generating a different data set, with each omic taken as a reference. (d) Postalignment filtering of samples with a higher alignment error than a predefined threshold. Sample C3015 in gray was discarded. (e) Biologically inspired Skeleton constraints imposed on training the DBNs computed by PALM. The biological assumption is that at the current time (*t_i_*), the expression of host genes (hexagons) and the environmental conditions (triangles) affect the abundances of microbial taxa (circles), which impacts the expression of microbial genes (diamonds), which in turn dictates the metabolites (squares) released, and which finally impacts the abundances of taxa in the next time instant (*t_i_*_+1_). These restrictions are flexible and can be modified by the user as input to the pipeline. (f) Learning a two-stage DBN structure and parameters, where nodes correspond to either host genes, environmental variables, taxa, genes, or metabolites. The figure shows two consecutive DBN time slices, *t_i_* and *t_i_*_+1_, where dotted lines connect nodes from the same time slice, referred to as intra-edges, and solid lines connect nodes between time slices, referred to as inter-edges. Biological relationships are annotated with the learned DBN parameters as either positive (green) or negative (red). Note that while this figure illustrates a general framework, in this study, we used only one static measurement (at baseline) for the host genes data.

We developed a computational pipeline (PALM), presented in Fig. 1, to process multi-omic microbiome data and infer their interactions. PALM first normalizes the data and then performs spline interpolation using continuous curves to enable imputation of missing time points and to overcome irregular sampling (Fig. 1b). We next temporally align the data to correct for the different progression rates of each individual (Fig. 1c) as well as filter out abnormal and noisy samples (Fig. 1d). Alignment can be performed using either of the data types, as we discuss below, and PALM extrapolates the transformation to the other omics types. Our dynamic Bayesian network (DBN) learning algorithm utilizes prior knowledge to constrain the resulting model, reducing overfitting and improving accuracy (Fig. 1e). These dynamic constraints can be customized in the form of an adjacency matrix. Using the imputed, aligned data, we train a DBN to model interactions within and between the different data types (Fig. 1f). Finally, we validate the model’s predictive ability and the edges using a curated list of taxon-gene and taxon-metabolite interactions.

### Resulting DBN models.

**FIG 2:**
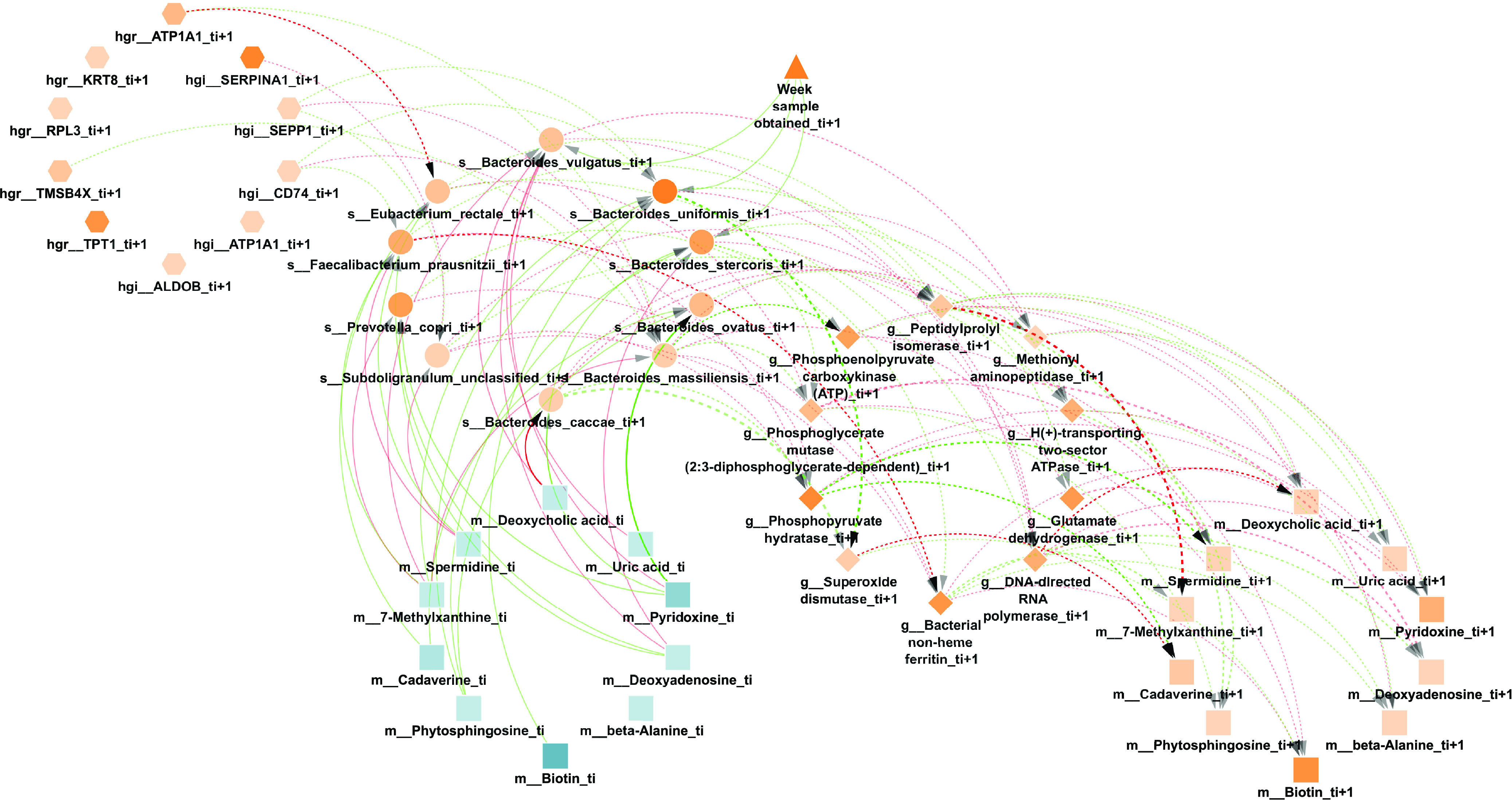
Learned DBN by PALM with the Skeleton constraints on the top 10 most abundant entities of each omic type and a maximum number of parents of 3. Nodes are either taxa (circles), genes (diamonds), metabolites (squares), host genes (hexagons), or environmental variables (triangles). The different node types have been grouped in different circles; their transparency is proportional to their average normalized abundance relative to that node type. While there are two consecutive time slices, *t_i_* (blue) and *t_i_*_+1_ (orange), nodes with no neighbors and self-loops were removed for simplicity. Dotted lines denote intra-edges (i.e., directed links between nodes in same time slice), whereas solid lines denote inter-edges (i.e., directed links between nodes in different time slices). Edge color indicates positive (green) or negative (red) temporal influence, and edge transparency indicates the strength of bootstrap support. Edge thickness indicates the statistical influence of the regression coefficient after normalizing for parent values, as described in reference 26.

We used the inflammatory bowel disease (IBD) cohort from the iHMP study (13), which followed 132 individuals over a year. These were profiled every 2 weeks, on average, for different omic types. The preprocessing steps included filtering, interpolation, temporal alignment, variable selection, and removal of subjects, with limited measured time points (see Materials and Methods for complete details). Based on these preprocessing steps, the resulting set used to train the model consisted of 50 individuals across 101 microbial taxa, 72 genes, and 70 metabolites. In addition, for each host, the model includes 40 gene measurements profiled at two sites (ileum and rectum) from a single sampled time point, and the only environmental variable considered was the week in which the sample was obtained. While PALM can use time series host expression data, the IBD study profiled individual expression levels only once at different sites. Thus, for the analysis presented here, we used the host expression profile as a static attribute. We used this data set to train multi-omic dynamic Bayesian models that provide information about interactions between taxa, genes, and metabolites and the impact of environmental variables and host transcriptomics on these entities over time. We used two sets of constraints: Skeleton and Augmented, depicted in Fig. S1 in the supplemental material and described in the section “Constraining the DBN structure,” below. The network with the complete IBD data set is presented in Fig. S2, with a total of two connections between the week of sample obtained and any other node, and Fig. S3, with no connections from the week variable, reinforcing the assumption that the system is in a steady state. For illustrative purposes of the capabilities of our methodology, we trained a DBN on a subset of the data set comprised solely of the 10 most abundant entities of each omic type, as shown in Fig. 2. Gene copy number effects can impact the observed expression levels. While this may be a problem for the normalization procedure, in general, such an impact is implicitly accounted for since our regression-based DBN uses taxon abundance as parents for the gene expression nodes.

10.1128/mSystems.01105-20.1 FIG S1 Adjacency matrix representation between microbiome entities for the two multi-omic frameworks used in this study: Skeleton (a) and Augmented (b). The figure highlights in red the added interactions in the Augmented framework compared to those of the Skeleton framework. Download FIG S1, PDF file, 0.06 MB. Copyright © 2021 Ruiz-Perez et al. 2021 Ruiz-Perez et al. https://creativecommons.org/licenses/by/4.0/ This content is distributed under the terms of the Creative Commons Attribution 4.0 International license.

10.1128/mSystems.01105-20.2 FIG S2 Two-stage DBN trained on the IBD data set by PALM with Skeleton constraints and a maximum number of parents of 3. Nodes are either taxa (circles), genes (diamonds), metabolites (squares), host genes (hexagons), or environmental variables (triangles). The different node types have been grouped in different circles; their transparency is proportional to their average abundance relative to that node type. While there are two consecutive time slices, *t_i_* (blue) and *t_i_*_+1_ (orange), nodes with no neighbors and self-loops were removed for simplicity. Dotted lines denote intra-edges (i.e., directed links between nodes in same time slice), whereas solid lines denote inter-edges (i.e., directed links between nodes in different time slices). The edge color indicates positive (green) or negative (red) temporal influence, and edge transparency indicates the strength of bootstrap support. Edge thickness indicates the statistical influence of the regression coefficient after normalizing for parent values, as described in reference 26. Download FIG S2, PDF file, 1.8 MB. Copyright © 2021 Ruiz-Perez et al. 2021 Ruiz-Perez et al. https://creativecommons.org/licenses/by/4.0/ This content is distributed under the terms of the Creative Commons Attribution 4.0 International license.

10.1128/mSystems.01105-20.3 FIG S3 Two-stage DBN trained on the IBD data set by PALM with Augmented constraints and a maximum number of parents of 3. Nodes are either host genes (hexagons), taxa (circles), genes (diamonds), or metabolites (squares). The different node types have been grouped in different circles, their transparency is proportional to their average abundance relative to that node type, and the two time slices were separated. Dotted lines denote intra-edges, whereas solid lines denote inter-edges. Edge color indicates positive (green) or negative (red) temporal influence, and edge transparency indicates the strength of bootstrap support. Edge thickness indicates the statistical influence of the regression coefficient after normalizing for parent values, as described in reference 26. Download FIG S3, PDF file, 1.3 MB. Copyright © 2021 Ruiz-Perez et al. 2021 Ruiz-Perez et al. https://creativecommons.org/licenses/by/4.0/ This content is distributed under the terms of the Creative Commons Attribution 4.0 International license.

In the DBN figures, each node represents either a bacterial taxon, a gene, a metabolite, or an environmental variable; directed edges represent inferred temporal relationships between these nodes. On the supporting website, we also provide a Cytoscape session with an interactive version of each network, together with the original files and a list of each edge’s learned weight for every network.

Figure S2 shows the full network learned by PALM, comprised of 284 nodes per time slice (101 microbial taxa, 72 genes, 70 metabolites, 40 host genes, and 1 environmental variable). To identify significant edges in the network, we applied bootstrapping, which involves rerunning the method 100 times, with each execution using a new data set created by randomly selecting, with replacement, as many subjects as there were in the data set. We next extracted all edges from all executions, resulting in 1,077 distinct directed edges (470 inter-edges and 607 intra-edges). Among all the 1,077 edges, we observed 362 (33%) negative interactions. Interestingly, a closer look at the learned DBN revealed that 79% (193 out of 243) of nodes with potential dependencies listed at least one negative interaction. Additionally, each edge is annotated with the percentage of bootstrap iterations in which it appears. Note that while there was considerable overlap between edges learned in each iteration, since we used the union of all the networks, the number of edges in the final network is larger than the number of possible edges for a single iteration (1,077 versus 284 × 3 = 852). While we focus mainly on the union, since it leads to more novel predictions, analysis of the intersection leads to similar statistical results. The learned DBN with the Augmented framework is shown in Fig. S3.

### Evaluating the learned DBN model.

**FIG 3:**
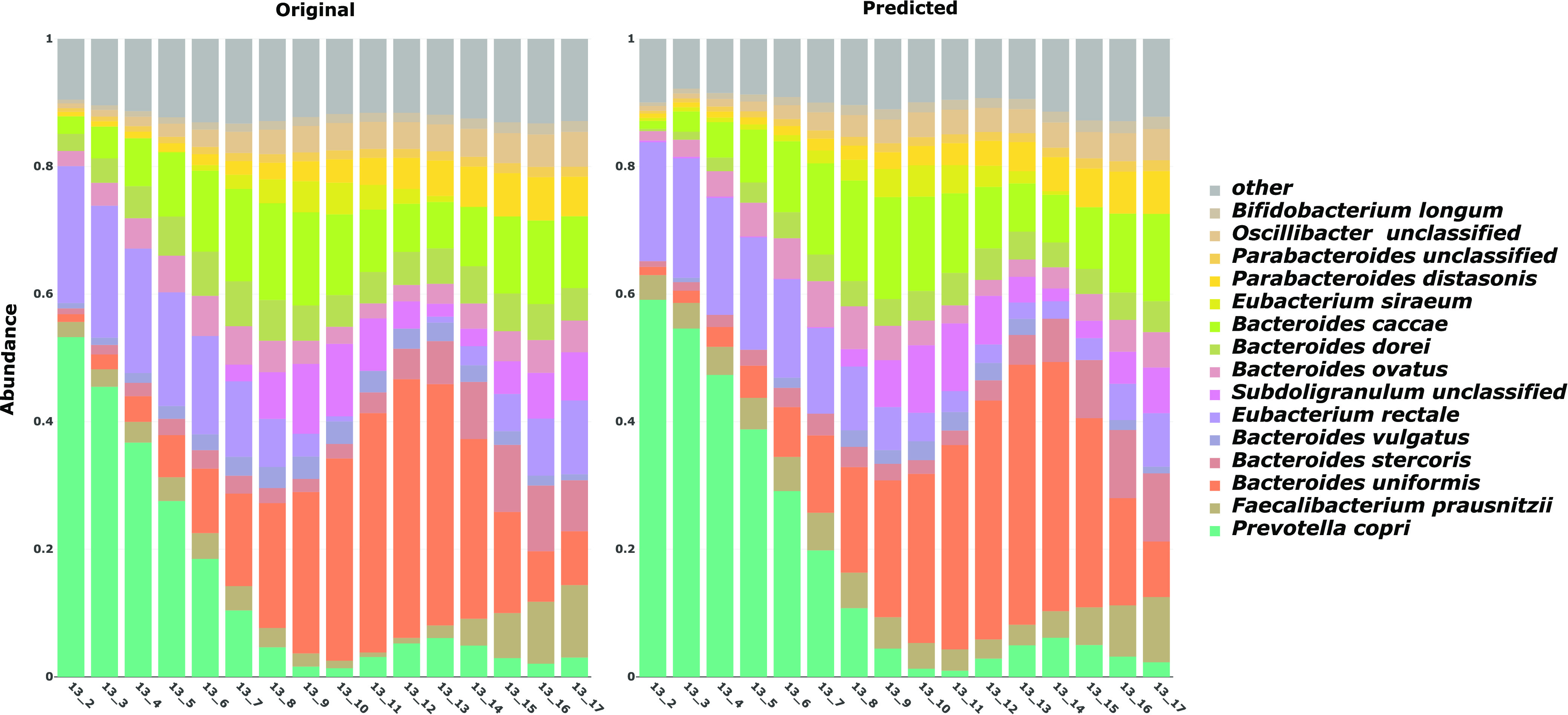
Comparison of observed versus predicted microbial composition trajectories. The figure shows the observed and predicted gene-aligned microbial composition trajectories for a representative aligned subject (C3013). The microbiota composition profile for this subject is comprised of the top 15 most abundant bacteria along with all remaining bacteria merged into the “other” category. The *y* axis corresponds to the relative abundance of each bacterium, while the *x* axis represents the original measured time point after alignment.

We first performed a technical evaluation of the learned DBN model and compared it to models constructed by other existing methods (26, 27). The performance of each model was evaluated through leave-one-out cross-validation, with the goal of predicting microbial composition using each learned model. Figure 3 represents the observed and predicted taxon compositions for subject C3013.

**FIG 4:**
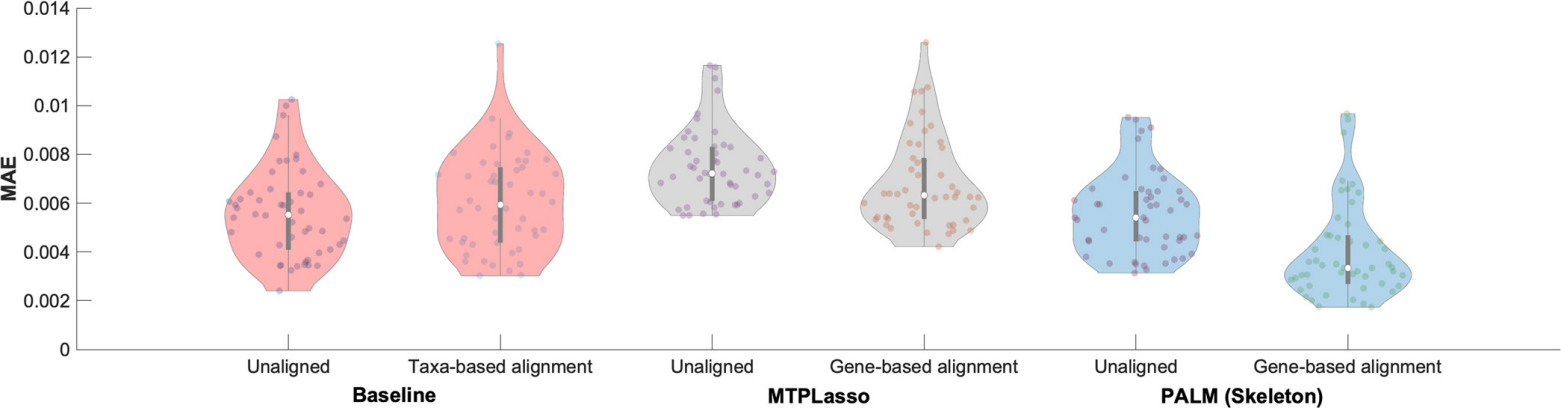
Comparison of average predictive accuracies between methods for the IBD data. The figure shows the MAE of our proposed DBN models against a baseline method using only metagenomic data and a previously published approach, MTPLasso, which models longitudinal multi-omics microbial data using a generalized Lotka-Volterra (gLV) model for a sampling rate of 2 weeks, which most closely resembles the originally measured time points. The figure also compares the performances of the methods on the unaligned and aligned data sets.

Additionally, we explored the effects of several different temporal alignments using taxa, genes, or metabolites. In each iteration, the whole longitudinal microbial abundance profile of a single subject was selected as the test set, and the multi-omics data from all other subjects were used for building the network and learning model parameters. Next, starting from the second time point, we used the learned model to predict an abundance value for every taxon in the test set at each time point using the previous and current time points. Finally, we normalized the predicted values in order to represent the relative abundance of each taxon and measured the average predictive accuracy by computing the mean absolute error (MAE) for the selected taxon in the network. This process of predicting microbial composition was repeated for different combinations of multi-omics training data (including metagenomics, metatranscriptomics, metabolomics, and host transcriptomics) on the aligned data sets, as well as unaligned data. A visual representation of the predicted trajectories for taxon- and gene-based alignment for subject C3028 is shown in Fig. S4a. The average MAE for the taxon predictions of PALM on the IBD data set for a sampling rate of 2 weeks using a gene-based temporal alignment is summarized in Fig. 4. Figure S5 shows the average MAE of PALM across different alignments based on taxa, genes, and metabolites. Finally, Fig. S6a and b show how MAEs vary for different maximum numbers of parents for Augmented and Skeleton, respectively.

10.1128/mSystems.01105-20.4 FIG S4 Comparison of predicted microbial composition trajectories and execution time. (a) Observed and predicted microbial composition trajectories for a representative aligned subject (C3028). The microbiota composition profile for this subject is comprised of the top 15 most abundant bacteria along with all remaining bacteria merged into the “other” category. The *y* axis corresponds to the relative abundance of each bacterium, while the *x* axis represents the original measured time point after alignment. The figure highlights the observed and predicted trajectories of this subject between taxon-based alignment (left) and gene-based alignment (right). We note that the aligned interval for gene-based alignment is stretched and shifted compared to that of the taxon-based alignment. For each alignment type, a DBN was trained with the Skeleton framework and a maximum number of parents of 3 and tested on the previously unseen C3028 subject. Gene-based alignment exhibits a lower prediction error (MAE = 0.0043) than taxon-based alignment (MAE = 0.0054). In this example, taxon-based alignment does a worse job at predicting low-abundance bacteria than gene-based alignment. (b) Observed and predicted microbial trajectories with the gene-aligned data using the log ratio normalization for subject C30130 (same as in Fig. 3). The abundance range is now from –*∞* to +*∞*. The predicted trajectories show remarkable similarities to the original data and trajectories similar to those of the compositional version from Fig. 3. Note that here the abundance of Faecalibacterium prausnitzii is zero because it was the taxon used for reference. (c) Experimentally, the execution time grows linearly with the number of parents. Note that while the times shown are for 1 repetition, the DBN figures shown are for 100. Download FIG S4, PDF file, 0.5 MB. Copyright © 2021 Ruiz-Perez et al. 2021 Ruiz-Perez et al. https://creativecommons.org/licenses/by/4.0/ This content is distributed under the terms of the Creative Commons Attribution 4.0 International license.

10.1128/mSystems.01105-20.5 FIG S5 Comparison of average predictive accuracies between methods on the IBD data sets aligned using taxon, gene, and metabolite data and a root mean square error (RMSE) comparison between MMvec and PALM when predicting metabolites. (a) MAE of PALM models (Augmented and Skeleton) against a baseline method and a previously published approach (MTPLasso) for a sampling rate of 2 weeks, which most closely resembles the originally measured time points. Although the baseline method uses only metagenomic data, gene- and metabolite-based alignments were generated using gene expression and metabolite intensity data, respectively. (b) Gene-aligned comparison between MMvec and PALM with Augmented and Skeleton restrictions for the whole data set (All). In addition, we compare MMvec with our learned DBNs with the Augmented constraints restricted to the data set with just taxa and metabolites (TM) in order to make the comparisons more fair. PALM greatly outperforms MMvec at the task at hand (*P* value = 4.10E–30 for a two-tailed paired *t* test against TM_Augmented) for every version tried. MMvec was executed with 100,000 epochs, five latent dimensions, a learning rate of 0.00001, and a batch size of 500, leaving all other parameters at the default. After we executed it five times, the execution with the lowest RMSE was chosen and compared against our method. The CV RMSE of the last 51 epochs (same as the number of subjects) are represented; because by this time the algorithm had already converged, the error variance of MMvec seems small. Download FIG S5, PDF file, 0.2 MB. Copyright © 2021 Ruiz-Perez et al. 2021 Ruiz-Perez et al. https://creativecommons.org/licenses/by/4.0/ This content is distributed under the terms of the Creative Commons Attribution 4.0 International license.

10.1128/mSystems.01105-20.6 FIG S6 MAE results as a function of the number of parents. (a and b) Average predictive accuracies of the learned DBNs with PALM under the Augmented and Skeleton frameworks, respectively, as a function of the maximum number of parents. For each parent choice, we show the MAE using the learned DBNs from unaligned and aligned data. Note that temporal alignments reduce MAE across all parent configurations, and the learned network for 3 parents shows the lowest average error over the others choices. It is worth highlighting that larger values for the maximum number of parents does not show an MAE improvement but significantly increases the run time for training the DBN structure, as shown in Fig. S4. Download FIG S6, PDF file, 1.2 MB. Copyright © 2021 Ruiz-Perez et al. 2021 Ruiz-Perez et al. https://creativecommons.org/licenses/by/4.0/ This content is distributed under the terms of the Creative Commons Attribution 4.0 International license.

We used this process to compare the multi-omics DBN strategy to the one that used only metagenomic data (26), referred to as Baseline on the unaligned and aligned IBD data, as well as MTPLasso (27), which models time series multi-omics microbial data using a gLV model. In both cases, we used the default setup and parameters, as described in the original publications. As shown in Fig. 4, our method outperforms Baseline and MTPLasso when gene expression data are used for temporal alignment of microbiome samples. Specifically, when gene expression data were used for alignment, the MAE significantly dropped to 4.01E–03, compared to an MAE of 6.03E–03, achieved using taxon alignment, as indicated by a one-tailed unpaired *t* test with the null hypothesis that the means are equal and the alternative hypothesis that the population mean of the method with gene expression-based alignment is less than the mean of the (Baseline) taxon-based alignment method (*P* value = 6.71E–07). Figure 4 also shows that gene-based alignment significantly outperforms unaligned data regardless of the underlying method used. Similarly, in the case of taxon- or metabolite-based alignment, Fig. S5 shows that our method outperforms MTPLasso when all microbiome entities are used in the model (taxon, 5.93E–03 versus 7.93E–03; metabolite, 5.82E–03 versus 7.97E–03). Moreover, Fig. S5 also shows that our method outperforms Baseline (taxon, 5.93E–03 versus 6.03E–03; gene, 4.01E–03 versus 4.19E–03; metabolite, 5.82E–03 versus 6.01E–03). Overall, our results suggest that gene expression data are more suitable for temporal alignment of multi-omics microbiome samples. This is consistent with previous findings which reported that technical noise dominates the abundance variability for nearly half of the detected taxa in gut samples (28). Therefore, we used gene-based alignment for the rest of the analysis, discussed next.

In addition, since PALM can easily be extended to predict other omic types, we predicted the metabolite concentration and compared our results with those of MMvec (19) (Fig. S5b). MMvec is the state of the art in metabolite prediction and uses neural networks for estimating interactions between microbes and metabolites through their cooccurrence probabilities. Our method significantly outperforms MMvec for all approaches tested (*P* value = 4.10E–30 for a two-tailed paired *t* test against the Augmented framework) at predicting metabolite abundances. We note that while MMvec aims to predict an entire metabolite abundance from the microbial read, PALM can also rely on metabolomics observations from previous time points for such predictions. Still, since MMvec is one of the only prior methods for linking metabolites and taxa, we compared our method to MMvec in Fig. S5b.

### Computationally validating predicted edges.

We compiled taxon-metabolite (*T*→*M*) and taxon-gene (*T*→*G*) databases and used those databases to validate the predicted edges and score each model. A *T*→*G* interaction was added to the database if any strain of taxon *T* has gene *G* in its genome according to the KEGG database. For *T*→*M*, we relied on the tool MIMOSA (29), which calculates the metabolic potential of each taxon for a particular data set. See Materials and Methods for complete details.

Each predicted interaction was either considered “validated” if it appears in the validation database or “not validated” if it was not found, but the parent and child nodes were part of the database. Interactions predicted between taxa and/or metabolites not included in the database were not used in this analysis. We compared the results between the learned DBNs by PALM using the Skeleton and Augmented constraints, as well as a random network. To generate the random network, we used the same nodes in the multi-omic network and assigned the same number of edges as in the learned DBN by randomly selecting a parent and child from the possible interaction list (Fig. S1). This was repeated 1,000 times, averaging the metrics over all random runs.

**FIG 5:**
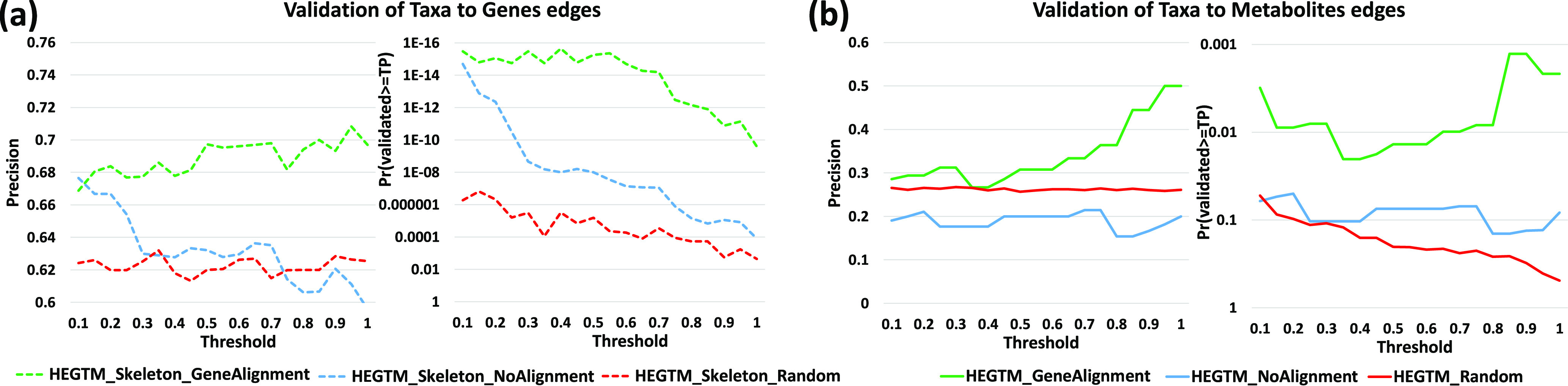
*In silico* validation results of the predictions of PALM for the IBD data set. The left part of each subfigure shows the precision (percentage of predicted edges that were validated), and the right part shows the probability of validating at least that many edges by chance (the *y* axis is in the reverse logarithm scale, so higher is better for both). The *x* axis represents the bootstrap value threshold that was used to select the edges included in the analysis. For example, for a threshold of 0.7, the score for edges that appear in more than 70% of the repetitions is shown. (a) Validation for *T*→*G* interactions (bacterial taxon expressing a gene). (b) Validation for *T*→*M* interactions (bacterial taxon consuming a metabolite).

Figure 5a shows the validation comparison for edges of the form *T*→*G*. The Skeleton constraints were used to train the networks. The learned DBN with the gene-aligned data set (green) was compared against a learned DBN with the data set that was not aligned (blue). As can be seen, the aligned data set results in networks that outperform the networks from the unaligned data and random networks, with the precision difference increasing as the threshold increases. This indicates that the bootstrap score for an edge can serve as a way to determine its likely accuracy.

Figure 5b shows the comparison for edges of the form *T*→*M*. For this, we can only use the network results from the Augmented constraints since no such edges are permitted when using Skeleton. Again, we observed better performance for the networks from aligned data than for the networks from unaligned data and random networks, with an improvement in performance for higher bootstrap thresholds. Note that for both *T*→*G* and *T*→*M*, the not-aligned network does not even outperform the random network, highlighting the importance of the alignment step.

Figure S7 shows the computational validation results of metabolite- and gene-based alignment and for various maximum numbers of parents for both restriction frameworks. These results highlight our choice of alignment and maximum-parent parameters. In addition, Fig. S8a shows the validation of the data set normalized using log ratios to circumvent bias in compositional data (30), and Fig. S4c shows the log ratio-normalized abundance trajectory prediction for the same subject as in Fig. 3. Finally, Fig. S8b shows the validation of learned DBNs with gene-based alignments between sampling rates of 14 days against 1 day using the Skeleton framework.

10.1128/mSystems.01105-20.7 FIG S7 *In silico* validation results with 100 bootstrap repetitions. (a and b) Graphs show the performance of different alignment types. (c and d) Variations in the number of parents used when training the networks. The left part of each panel shows the precision (the percentage of predicted edges that were validated), and the right part shows the probability of validating at least that many edges by chance (the *y* axis is in reverse logarithm scale, so higher is better for both). The *x* axis represents the bootstrap value threshold that was used to select the edges included in the analysis. For example, for a threshold of 0.7, the score for edges that appear in more than 70% of the repetitions is shown. The dashed lines (*T*→*G* interactions) were trained using the Skeleton constraints, and the solid lines (*T*→*M* interactions) were trained using the Augmented constraints, because the *T*→*M* edges are not allowed directly in Skeleton. (a) Validation for *T*→*G* interactions (bacterial taxon expressing a gene), varying the alignment reference used. No alignment barely does better than the random baseline, followed closely by the metabolite-based alignment. Taxon-based alignment has a slightly better precision than gene-based alignment, but the latter has a much better probability score than the former. (b) Validation for *T*→*M* interactions (bacterial taxon consuming a metabolite), varying the alignment reference used. Taxon- and metabolite-based alignments have a lower precision than the random baseline but a better probability score. (c) Validation for *T*→*G* interactions (bacterial taxon expressing a gene), varying the maximum number of parents allowed. Learning with 3 parents has a much better precision than with 4 and 5 and a similar probability score. (d) Validation for *T*→*M* interactions (bacterial taxon consuming a metabolite), varying the maximum number of parents allowed. Learning with 3 parents has a better precision than with 4 and 5 for small and big thresholds. Learning with 5 parents has a better probability score for low thresholds, but it seems that it is by chance, because as the threshold becomes more stringent, it quickly fares worse, while 3 parents overtakes 4 parents by a small percentage. Download FIG S7, PDF file, 0.9 MB. Copyright © 2021 Ruiz-Perez et al. 2021 Ruiz-Perez et al. https://creativecommons.org/licenses/by/4.0/ This content is distributed under the terms of the Creative Commons Attribution 4.0 International license.

10.1128/mSystems.01105-20.8 FIG S8 *In silico* validation results for different sampling rates and normalizations. (a and b) Comparison of relative abundance normalization and the log ratio normalization. (c and d) Comparison of sampling rates of 14 days (14d) and 1 day (1d). The left part of each panel shows the precision (percentage of predicted edges that were validated), and the right part shows the probability of validating at least that many edges by chance (the *y* axis is in reverse logarithm scale, so higher is better for both). The *x* axis represents the bootstrap value threshold that was used to select the edges included in the analysis. (a) Validation for *T*→*G* interactions (bacterial taxon expressing a gene). Alignment seems to be helpful for the relative abundance normalization but not for log ratio normalization, the performance of which is similar to that of the relative abundance no-alignment approach. Our selected method of relative abundance gene alignment outperforms the others for both precision and probability. (b) Validation for *T*→*M* interactions (bacterial taxon consuming a metabolite). The alignment version does better than the no-alignment method, for both normalization methods. Relative abundance normalization exhibited a better precision and statistical significance than log ratio normalization, regardless of alignment type. This difference closes in for gene alignment as the *x* axis grows. However, we emphasize that differential abundance analysis based on relative abundance may be problematic since it relies on very strong assumptions about overall taxon abundance similarity between time points. (c) Validation for *T*→*G* interactions (bacterial taxon expressing a gene). Both the precisions and the probabilities are very similar for both sampling rates, with 14-day sampling outperforming 1-day sampling by a small amount. (d) Validation for *T*→*M* interactions (bacterial taxon consuming a metabolite). The 14-day sampling rate option clearly outperforms the smaller sampling rate data set, with both better precision and better probability. Download FIG S8, PDF file, 0.8 MB. Copyright © 2021 Ruiz-Perez et al. 2021 Ruiz-Perez et al. https://creativecommons.org/licenses/by/4.0/ This content is distributed under the terms of the Creative Commons Attribution 4.0 International license.

### Biological validation experiments.

We performed experiments to validate a few of the interactions predicted by the DBNs. We focused on edges of the form *M*→*T*, i.e., edges where a metabolite is predicted to impact the abundance of a bacterial taxon. Such edges imply that the metabolite *M* promotes (or represses, depending on the sign) the growth of the bacterial taxon *T* under appropriate growth conditions.

We first sorted all predicted *M*→*T* interactions based on their confidence (product of normalized weight and bootstrap score). Next, we selected some of the top edges to validate taking into account the availability of the metabolites and taxa and the laboratory resources for growth experiments at our disposal. See the section “Laboratory validations of (metabolite-to-taxon) edges,” below, for details on this process. Based on these considerations, we focused on two common model organisms, namely, Pseudomonas aeruginosa and Escherichia coli, and picked from the top predictions those involving any of these two taxa for validation. In addition, we trained an independent densely sampled network (sampling rate of 1 day) to address the possible disconnect between the sampling rates of the DBN and the experiments. The results show that the learned networks are consistent when the much denser sampling is used. Specifically, the Skeleton gene alignment networks shared 372 out of 671 (55%) interactions, with a bootstrap score of ¿= 0.5 (given the sparsity of the networks, this is a significant overlap). Only 10 bootstrap repetitions were used due to the computational resources needed. More importantly, all three positive controls tested in the wet lab were also found in the unaligned and aligned (metabolite-based) networks using the Skeleton framework. Furthermore, two out of the three positive controls were also found in the learned networks from gene- and taxon-based alignments. Interestingly, none of the densely sampled networks learned the negative-control interaction.

- 4-Methylcatechol (4-MC) → Escherichia coli
- 4-Hydroxyphenylacetate (4-HPA) → *Escherichia* unclassified
- d-Xylose → *Pseudomonas* unclassified

**FIG 6:**
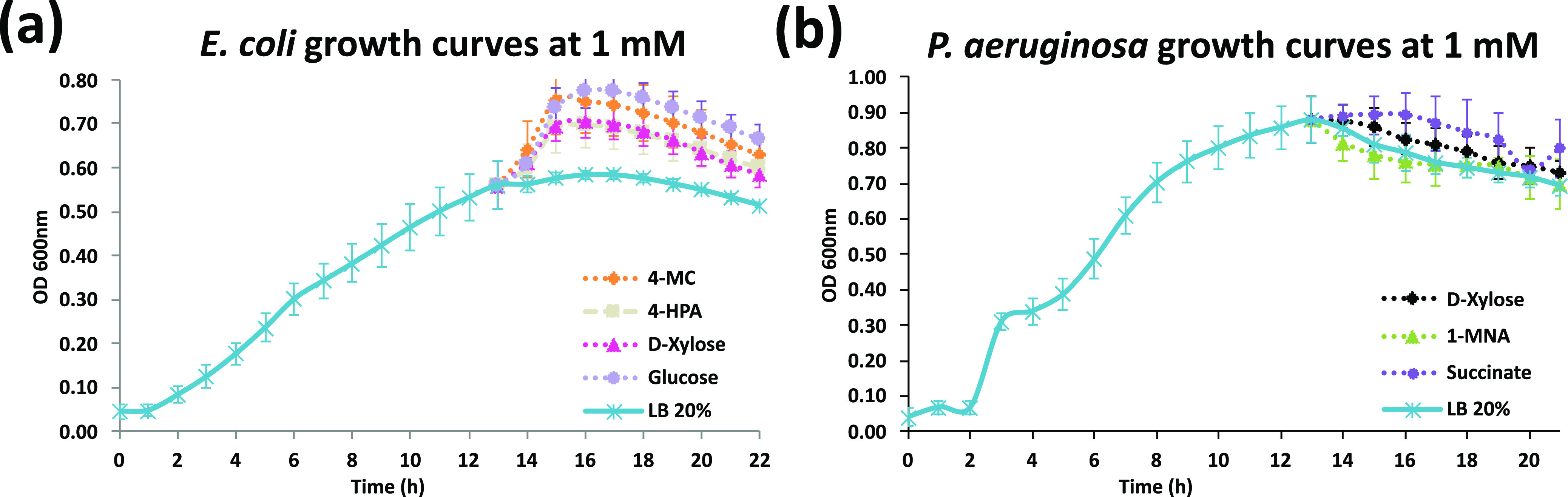
Growth curves at 1 mM. In this figure, different metabolites were introduced at a 1 mM concentration at the end of the exponential phase (0 to 14 h). The figure shows the growth curves after all data points were averaged over 10 replicates. (a) E. coli, with glucose and d-xylose as positive controls. (b) P. aeruginosa, with succinate as a positive control and 1-MNA as a negative control.

Standard lab strains, P. aeruginosa PAO1 (31) and E. coli HB101 (32), were used in the laboratory experiments. The choice of chemicals used to verify was somewhat limited by commercial availability. A standard Luria-Bertani (LB 20%) culture medium was used to measure the bacterial growth curve, expressed as bacterial density (optical density at 600 nm [OD_600_]), in the absence and presence of metabolites. Metabolites were added at the stationary phase when the bacteria were multiplying very slowly, mimicking biofilm growth (33). As positive controls, the preferred carbon sources of E. coli and P. aeruginosa, glucose and succinate, respectively, were chosen. Figure 6 shows the resulting growth curves of the microbes before and after addition of the metabolites and a control case without addition of the metabolites (LB 20%). Confirming the predictions of our networks, d-xylose significantly enhanced P. aeruginosa, and 4-HPA and 4-MC significantly increased E. coli growth. Regarding the controls, as expected, d-xylose and glucose enhanced E. coli, and succinate enhanced P. aeruginosa, whereas the negative control 1-methylnicotinamide (1-MNA) did not.

The *P* values for all observations can be seen in Table 1, where a two-tailed paired *t* test was executed for the three time points with the highest difference from the baseline. For more details on the experimental settings, please refer to the section “Laboratory validations of (metabolite-to-taxon) edges,” below, as well as Fig. S9 for growth results at a lower concentration of 0.2 mM.

**TABLE 1:**
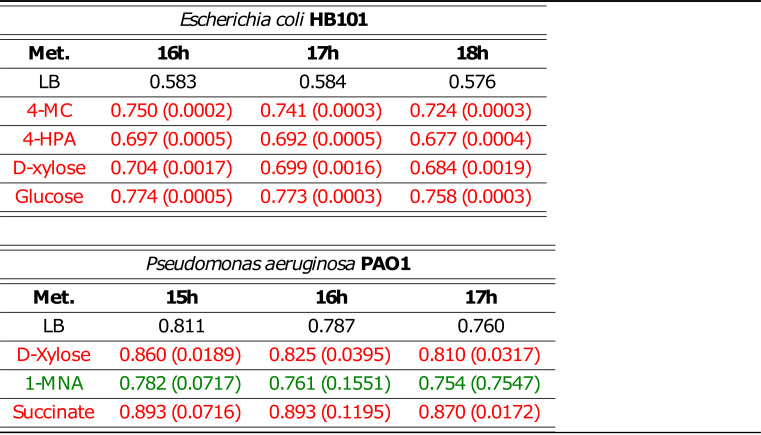
Effect of 1 mM metabolites on bacterial cell density^*a*^

a Taxon density appears in black (OD_600_), while the *P* values are inside parentheses. Red *P* values represent a significant difference from LB 20% values (*P* < 0.05). Green *P* values represent a nonsignificant difference from LB 20% values. Met., metabolites.

10.1128/mSystems.01105-20.9 FIG S9 Growth curves and metabolite effect (0.2 mM). This figure shows the result of introducing different metabolites at a 0.2 mM concentration at the end of the exponential phase (up to 14 h). (c and d) Taxon density appears in black, while the *P* values are inside parentheses. Red *P* values represent a significant difference from LB values (*P* < 0.05). Green *P* values represent a nonsignificant difference from LB values. (a and c) Growth curve and the growth results, respectively, for E. coli, with glucose and d-xylose as positive controls. (b and d) Growth curve and the growth results, respectively, for P. aeruginosa, with succinate as a positive control and 1-MNA as a negative control. Download FIG S9, PDF file, 0.4 MB. Copyright © 2021 Ruiz-Perez et al. 2021 Ruiz-Perez et al. https://creativecommons.org/licenses/by/4.0/ This content is distributed under the terms of the Creative Commons Attribution 4.0 International license.

## DISCUSSION

Previous microbiome studies focused primarily on metagenomics sequence data. More recent data sets are much richer, notably including host and bacterial gene expression and metabolomics data. The ability to integrate these multi-omics with longitudinal data remains a major challenge for microbiome analysis.

Here, we have presented PALM, a new approach based on a temporal normalization using continuous curve alignment, followed by DBN modeling. Our method first represents each time series using continuous curves and then aligns them using a reference time series. Next, we sample the aligned curves uniformly and train a DBN model that combines data from taxa, host genes, bacterial genes, and metabolites. Edges in the DBN represent predicted interactions between the entities and can be used to explain changes in the microbiome over time.

Applying our methods to data from IBD patients, we show that multi-omics DBNs can successfully predict taxon abundance at future time points, thus improving on models that do not use all available data and on previous methods developed for modeling temporal taxon interactions. We curated validations for taxon-to-metabolite and taxon-to-gene interactions; edges predicted by the learned DBNs significantly intersect these interactions. Finally, we experimentally tested and validated select predictions of metabolite-to-taxon relationships. We have ignored the case-control structure of the IBD data set for this work, but the framework can easily be used for that purpose either by training different models for each disorder or by adding a “diagnosis” node to the network and studying its outgoing edges. To address any concern regarding the learning test independence resulting from the alignment step, we highlight that the predicted trajectory is evaluated using 101 taxon profiles, while only a subset of them (9 out of 101) participated in the temporal alignment step. More importantly, in the results presented in this paper, we did not rely on taxon-based alignment. Instead, we used gene expression for the temporal alignments and computed the MAE from predicting microbial composition trajectories. While there may be correlation between taxa and genes, this is much weaker than using the taxon itself for the alignment, and so the results that we present do show that alignment improves the ability to predict microbiota composition.

Microbiome interaction databases are critical for evaluating learned DBNs but appear to be incomplete. More complete databases of validated interactions would help validate computational methods for this task. The laboratory validations show a viable way to validate some of the interactions. However, they may also be improved by attempting to re-create more realistic conditions for the experiments and may be enhanced to validate other omics observations as well. Our models suggest that certain metabolites can be used as predictors of the abundance of taxa. Our interpretation is that bacteria consume these metabolites, which causes fluctuations in their abundances, but other indirect effects may also be possible.

Comparing DBNs constructed using different omics data allows for an important kind of inference (Fig. S10). According to this premise, in the DBN built using only metagenomics data, the edge Streptococcus parasanguinis → *Pseudomonas* unclassified appears with a high confidence (bootstrap score of 1). In the multi-omic DBN, the following chain of interactions can be found: Streptococcus parasanguinis (T) → RNA polymerase (G) → d-xylose (M) → *Pseudomonas* unclassified (T). It is important to note that though DBN edges may not imply causal relationships, the *in silico* validation process described in this paper supports the above relationships. Finally, d-xylose → *Pseudomonas* unclassified was validated experimentally (see “Biological validation experiments,” above). Thus, comparing DBNs before and after adding additional multi-omics data can “unroll” and “explain” relationships between taxa.

10.1128/mSystems.01105-20.10 FIG S10 The edge Streptococcus parasanguinis → *Pseudomonas* unclassified on the bottom gets explained when multiomic data are added (T stands for a data set with just taxa). In the multi-omic network, that interaction gets replaced by Streptococcus parasanguinis (T) → RNA polymerase (G) → d-xylose (M) → *Pseudomonas* unclassified (T). Download FIG S10, PDF file, 0.05 MB. Copyright © 2021 Ruiz-Perez et al. 2021 Ruiz-Perez et al. https://creativecommons.org/licenses/by/4.0/ This content is distributed under the terms of the Creative Commons Attribution 4.0 International license.

Our alignment and DBN methods are implemented in Python and Matlab. The source code and data set used can be obtained from the link in the section “Data availability,” below, to reproduce the findings of this paper, together with the networks learned and interactions predicted, sorted by relevance.

## MATERIALS AND METHODS

Below, we describe the computational pipeline PALM, developed to integrate and model the interactions between the different types of omics.

### Data.

To test PALM’s proposed analysis pipeline, which combines temporal alignment with Bayesian network learning and inference for multi-omics microbiome data, we used the inflammatory bowel disease (IBD) cohort from a study that included 132 individuals across five clinical centers (13). During a period of 1 year, each subject was profiled (biopsy specimens, blood draws, and stool samples) every 2 weeks, on average. This yielded temporal profiles for metagenomes, metatranscriptomes, proteomes, metabolomes and viromes across all subjects. Specifically, a taxon abundance profile is a sequence of measurements capturing the abundance levels of a specific microbial taxon over time. Although the metatranscriptomics data summarized functional profiling via HUMAnN2 (34) with species-specific and species-agnostic quantification of gene families, EC enzyme modules, and pathways, we focused solely on metabolic enzyme quantification (i.e., of EC enzyme modules) at the community level. We note that the proposed framework can be used to integrate the full metatranscriptomics data (gene families, EC enzyme modules, and pathways) as well as other omics data (e.g., viromics); however, given the complexity of the current data sets that we have modeled here, we have restricted the analysis to only EC enzyme modules in this paper. Additionally, for each subject, host- and microbe-targeted human RNA sequencing was yielded from biopsy specimens collected at the initial screening colonoscopy, with patients sampled at three locations (colon, ileum, and rectum); however, only ileum and rectum data were used in this study, as colon data were missing for 82% of the subjects analyzed. All data sources are fully described and available at https://ibdmdb.org/.

Each data set was associated with the week when it was sampled, except for the host transcriptomics, for which only a single biopsy specimen was obtained at each location (ileum and rectum). Therefore, host transcriptomics data were used as static variables in the DBN.

### Data preprocessing.

First, for each subject, the different omic profiles (taxon, gene, and metabolite) were normalized separately such that each omic type sums up to 1. In order to account for the common pitfalls when comparing relative abundances across samples, as highlighted by recent studies (30, 35, 36), we also separately normalized microbial taxon data using log ratios, which have been shown to circumvent bias in compositional data (30). Specifically, we used Faecalibacterium prausnitzii as the reference species. Next, for each individual with *n *> 1 different longitudinal microbial samples, *s*_1_,⋯,*s*_n_, we computed the additive log ratio transformation (37) of the *i*-th sample as

$$\log\frac{s_{i}}{s_{1}},\mathrm{for}\;i=2,\cdots ,n.$$

Additionally, RNA-seq data from the ileum and rectum of each host were analyzed using DESeq2 (38) and the count of transcripts per kilobase million (TPM). As a proof of concept, for each body site, we selected the top 20 genes with the highest variance across all subjects. We note that this set of genes is limited, as previous studies have reported over 1,000 differently expressed genes for IBD individuals at these locations compared to individuals without IBD (13). In the case of metabolomics data, metabolites without an HMDB correspondence were removed. Then, we filtered out metabolites for which the mean intensity was less than 0.1% or had zero variance from the originally sampled time points. Next, we performed temporal alignment of time series data from individuals, as described in the work of Lugo-Martinez et al. (26). For this, we needed to represent each discrete time series using a continuous function. Here, we used B-splines for fitting continuous curves to the time series multi-omic data profiled from each subject, including the microbial composition, gene expression, and metabolic abundance. To improve the accuracy of the reconstructed profiles, we removed any sample that had fewer than five measured time points in any of the multi-omics measurements. Although the IBD data are composed of 107 individuals with taxonomic profiles, 77 individuals with gene expression profiles, and 80 individuals with metabolic profiles, and each omic profile has at least 5 longitudinal measurements, there are exactly 62 individuals whose trajectories have at least 5 measured time points for all three omic types. This set is further reduced to 51 individuals when cross-referenced with the gene expression profiles derived from host transcriptomics data. Therefore, the final set is comprised of 51 individual multi-omic time series used for further analysis.

### Temporal alignments.

Given longitudinal samples from different subjects, we cannot expect that the rates at which various multi-omics levels change would be exactly the same between these individuals (39). To facilitate the analysis of such longitudinal data across subjects, we first aligned the time series from the microbiome samples using the microbial composition profiles. As described earlier, these alignments use a linear time transformation function to warp one time series into a common, representative sample time series used as the reference (26). While prior alignment methods relied on taxon information, when multi-omics data are available, PALM can use other genomic information for the alignment. Specifically, here we also tested the use of gene expression and metabolite abundance profiles for determining accurate alignments of patients. As we show, by using a better omics data type, the resulting DBNs can more accurately capture and predict taxon-metabolite and taxon-gene relationships.

For each omics data type (i.e., taxa, genes, or metabolites), we selected an optimal reference sample from the 51 time series as follows: we generated all possible pairwise alignments between them and selected the time series that resulted in the least total overall error in the alignments. We then searched for abnormal and noisy samples from the resulting set of alignments as follows: (i) computed the mean (*μ*) and standard deviation (*δ*) of the alignment error and (ii) removed all samples from an individual whose alignment error exceeded μ+(2×δ), as previously described in the study by Lugo-Martinez et al. (26). However, we did not remove any of the 51 time series samples, as none of the aligned profiles displayed an alignment error satisfying these constraints across all three omic types. Figure 1a to d show the overall alignment process of Bacteroides dorei from the taxon-based alignment perspective.

Given an individual’s warped/aligned time series over a specific omic type, the other multi-omics data were incorporated as follows: the same transformation applied to the aligned sample was applied to all the complementary multi-omics time series data. The resulting set used for the modeling was comprised of 50 individual-wise heterogeneous alignments involving 101 microbial taxa, 72 genes, and 70 metabolites. This smaller number of attributes was used because learning a Bayesian network is computationally intractable (40, 41) and is unlikely to have a run time that is polynomial in the number of features; however, there is no imposed restriction on the number of features.

### Dynamic Bayesian network models.

Using the aligned time series multi-omics data, we next trained graphical models that provide information about the relationships between the different omics (taxa, genes, metabolites, host genes) and environmental (exogenous) variables. In PALM, we extend the DBN model proposed in the work of Lugo-Martinez et al. (26) to account for multi-omics microbiome data with the goal of inferring the temporal relationships between the heterogeneous entities in a microbial community. A DBN is a directed acyclic graph where, at each time slice, nodes correspond to random variables of interest (e.g., taxon abundance, gene expression, age, etc.) and directed edges correspond to their conditional dependencies in the graph. These edges are defined as either intra-edges, connecting nodes from the same time slice, or inter-edges, connecting nodes between consecutive time slices. In our DBN model, only two slices are modeled and learned, as shown in Fig. 1e.

In PALM, our DBN models encode five types of nodes: (i) taxon abundance, (ii) gene expression, (iii) metabolite concentration, (iv) host gene expression, and (v) sample metadata information. The first three types represent continuous variables, whereas the last two types can be either discrete or continuous. For our DBNs, we use the formalism of conditional Gaussian Bayesian networks (42) to take advantage of its ability to seamlessly integrate discrete and continuous variables in a single probabilistic framework. However, since all DBNs presented in this paper use only continuous variables, we excluded any reference to discrete variables in the joint probability distribution. Formally, let Θ denote the set of parameters for the DBN and *G* denote a specific network structure over continuous variables (denoted as Ψ) in the multi-omics microbiome study. The joint distribution *P*(Ψ) can be decomposed as

$$P(\Psi )=\prod_{x\in \Psi }f[x\;|\;Pa^{G}(x)],$$

where *f* denotes a linear Gaussian conditional density over continuous variables and ***Pa**^G^*(*x*) denotes the set of parents for the variable *x* value in *G* (26, 43). In particular, continuous variables are modeled using a Gaussian model with the mean set based on a regression model over the set of continuous parents, as follows

$$f(x\;|\;u_{1},\cdots ,u_{k})∼N(\beta_{0}+\sum_{i=1}^{k}\beta_{i}\times u_{i},\sigma^{2}),$$

where *u*_1_,…,*u_k_* are continuous parents of *x*, *β*_0_ is the intercept, β_1_,…,β_*k*_ are the corresponding regression coefficients for *u*_1_,⋯,*u_k_*, and *σ*^2^ is the standard deviation. This Gaussian regression model is appropriate for modeling errors and is at least partially a way to deal with both normalization impact and measurement noise. As highlighted in Fig. 1e, the conditional linear Gaussian density function for variable $T_{1}^{t_{i+1}}$ denoted as $f(T_{1}^{t_{i+1}}\;|\;T_{1}^{t_{i}},M_{1}^{t_{i}},E_{1}^{t_{i+1}},E_{2}^{t_{i+1}})$ is modeled by

$$N(\beta_{0}+\beta_{1}\times T_{1}^{t_{i}}+\beta_{2}\times M_{1}^{t_{i}}+\beta_{3}\times E_{1}^{t_{i+1}}+\beta_{4}\times E_{2}^{t_{i+1}},\sigma^{2}),$$

where Θ={β_1_,β_2_,β_3_, σ^2^} is the set of DBN model parameters. Here, we infer the parameters, Θ, by maximizing the likelihood of the longitudinal multi-omics data, *D*, given our regression model and known structure, *G*.

The problem of training the DBN is expressed as finding the optimal structure and parameters,

$$\underset{\Theta ,G}{\max}P(D\;|\;\Theta ,G)P(\Theta ,G)=\underset{G}{\max}P(D,\Theta \;|\;G)P(G),$$

where *P*(*D* | Θ,*G*) is the likelihood of the data given the model. Since the likelihood of a structure increases as the number of edges increases, one must effectively find the structure that maximizes the likelihood of the data while penalizing overly complex structures. As in the study by Lugo-Martinez et al. (26), we maximized *P*(*D*,Θ | *G*) for a given structure, *G*, using maximum log-likelihood estimation (MLE) combined with a Bayesian information criterion (BIC) score, defined as

$$\text{BIC}(G,D)=\log P(D\;|\;\Theta ,G)-\frac{\left|\Theta \right|}{2}\log|D|,$$

where |Θ| is the number of DBN model parameters in structure *G* and |*D*| is the number of observations in *D*. This approach enables an effective way to search over the set of all possible DBN structures while favoring simpler structures. Furthermore, this approach has been shown to outperform Bayesian-Dirichlet scores, which require prior knowledge and can be sensitive to parameters and improper prior distributions (26, 44, 45).

### Constraining the DBN structure.

An important innovation in PALM lies in the structure constraining of the network to conform to our proposed metabolic framework, which ensures the desired flow of interactions. These constraints (in the form of a matrix received as an input to the function) allow edges only between certain types of nodes, highly reducing the complexity of searching over possible structures and preventing overfitting. Note that these constraints can easily be modified by the user, such as by adding more data types or different restrictions in the input file containing the adjacency matrix. Specifically, we allowed intra-edges from environmental and host transcriptomics variables to microbial taxon (abundance) nodes, from taxon nodes to gene (expression) nodes, and from gene nodes to metabolite (concentration) nodes. All other interactions within a time point (for example, direct gene to taxa) were disallowed. We also allowed inter-edges from metabolites to taxon nodes in the next time point, and self-loops from any node, $A_{1}^{t_{i}}$, to $A_{1}^{t_{i+1}}$, except for environmental or host transcriptomics variables for which no incoming edges were allowed (host genes were measured at only a single time point, so no incoming temporal edges were allowed for them). These restrictions referred to as the Skeleton and depicted in Fig. S1a reflect our understanding of the basic ways that the different entities interact with each other; i.e., environmental and host gene expression variables are independent variables, taxa express genes, which are involved in metabolic pathways, and finally, the metabolites impact the growth of taxa (in the next time slice).

We also trained DBNs using a less constrained framework, referred to as Augmented, as shown in Fig. S1b. Unlike Skeleton, the Augmented framework also allowed direct edges between taxa and metabolites to account for cases where noise or other issues related to profiling of genes can limit our ability to indirectly connect taxa and the metabolites that they produce. Figure S1 summarizes each framework in the form of an adjacency matrix. Note that other constraints, such as requiring that taxa connect only to genes present in their genome, were not imposed since genomics reference databases are not always complete and so they may lead to missing key interactions.

We used a greedy hill-climbing approach for structure learning, where the search is initialized with a network that connects each node of interest at the previous time point to the corresponding node at the following time point. Next, nodes are added as parents of a specific node via intra- or inter-edges, depending on which valid edge leads to the largest increase of the log-likelihood function beyond the global penalty incurred by adding the parameters as measured by the BIC score approximation.

Every network was bootstrapped by randomly selecting with replacements of as many subjects as in the data set and training a different network 100 times. Although we explore multiple values as the maximum number of possible parents for each node (see Fig. S6 and S9, S10, and S11 for results with different maximum numbers of parents), unless otherwise stated, the maximum number of possible parents was fixed at 3. The networks were then combined, and the regression coefficients of the edges were averaged. Each edge was also labeled with the bootstrap support (percentage of times that the edge appears). Each repetition was set to run independently on a separate processor using MATLAB’s Parallel Computing Toolbox. Other parallel implementations include parallelizing the cross-validation computation of the inference error and each independent alignment error calculation using Python’s Parallel library.

### Validating DBNs.

A major challenge in building models of biological interactions lies in developing methods to validate them and in providing confidence measures. Since DBNs are generative models, one approach is to predict time series using previous time points and thus to achieve cross validation (26). Such technical validations, while informative, can be thought as of “black-box” validation and do not shed light on the accuracy of specific edges and interactions predicted by the model in which we are interested.

We broadly discuss approaches to validate the types of edges present in the DBN (Fig. 1e), which are the parameters learned by the model and hence closer to “white-box” validation. Edges from taxa to genes can be circumstantially validated by verifying that (i) the taxon presence is guaranteed by its nonzero abundance, (ii) the taxon genome has the gene, and (iii) the gene is expressed. PALM, therefore, handles this using the *in silico* validation strategies mentioned below. Similarly, edges from genes to metabolites or taxa to metabolites could potentially be validated.

The challenge is in validating edges from metabolites to taxa, for which an *in silico* approach is unlikely to work since no such database has been compiled to the best of our knowledge. In “Laboratory validations of (metabolite-to-taxon) edges,” below, we propose a validation approach involving laboratory experiments.

### *In silico* validation of DBN edges.

*In silico* validations of DBN edges are handled by verifying the information against a database of known interactions between taxa to genes and/or taxa to metabolites. Unfortunately, no such comprehensive database exists. For example, highly curated databases, such as the HMDB (46) or MetaCyc (47) database, or the findings of the large-scale study of Maier et al. (48) turned out to be inadequate since the intersection of their contents with the species and metabolites in our networks was too small.

To assist in the validation of taxon-metabolite (*T*→*M*) edges in our networks, we relied on the tool MIMOSA (29). MIMOSA calculates the metabolic potential of each species, i.e., the ability of a species to produce a metabolite under the conditions of the data set. The list of all taxon-metabolite pairs from our DBNs that resulted in a positive score in MIMOSA was used as a validation database.

For taxon-gene (*T*→*G*) validations, we used the KEGG database to build a validation database of bacterial taxa and the genes present in their genomes. To keep this database small, we used only taxa and genes present in our network. If multiple strains were available for a bacterial species, then all genes from each strain were aggregated. The one-time creation of a local validation database also speeded up our computations considerably.

To calculate the statistical significance of validated interactions compared to a null model, a Poisson-binomial distribution test was executed to test if the interactions were expected to be validated by chance. The main reason that a simple binomial test cannot be performed is the differences in the in-degree distribution between different nodes in the validation set (essential metabolites or genes would have a high probability of being connected to any given bacterium in the validation database). Because some nodes have many more validated interactions than others, a uniform model for each edge does not accurately capture the null probability of selecting such an edge. This was solved with the function ppoisbinom from the R package poisbinom (49), which gives the cumulative distribution function of the probability of validating by chance at least as many interactions as the number of true positives. For this calculation, each possible interaction has a different probability of being selected (based on the validation database). The validation precision of the network was also calculated as the percentage of validated interactions from the ones predicted, even though this homogeneous metric ignores the differential significance of each interaction. We did not calculate the validation recall because in many cases a false positive cannot be distinguished from a true interaction that was simply not recorded in the database and may lead to misleading scores.

### Laboratory validations of (metabolite-to-taxon) edges.

Wet-lab experiments were carried out to validate predicted *M*→*T* interactions. Testing each such edge is not a feasible proposition. We first sorted all predicted *M*→*T* interactions based on their confidence, which we defined as the value of |normalize(weight)| × bootstrap. We applied this operation to the three parents (Skeleton for the gene-aligned and no-alignment networks). The normalization was performed to counteract the differences in the abundances between the parent and child nodes according to reference 26. We narrowed it down to edges that involved the species P. aeruginosa or E. coli because of the ready availability of these species and the expertise and facilities available to us in our laboratories. Then, we combined and sorted all interactions from the gene alignment and no-alignment networks and selected the top interaction involving P. aeruginosa and the top two interactions involving E. coli to validate. The full list of interactions along with their confidence scores can be seen in the Networks folder of the GitHub page. For positive controls, we selected metabolites known to enhance growth, and as a negative control, we selected one metabolite that was not connected to the taxon in any of our learned networks.

The goal of the experiments was to validate an *M*→*T* edge by studying the impact of the metabolite *M* on the growth of taxon *T*. While the experimental setup does not re-create the conditions of the interaction in the microbiome, we consider this an important step in the right direction. As with the *in silico* validations, the laboratory validation confirms that the inferred interaction is a strong possibility. We selected three predicted interactions involving readily available bacteria and metabolites from the generated networks. The experiments were performed by growing relevant taxa in isolation and adding the relevant metabolite to measure its impact on growth. These metabolites were expected to positively impact the growth of the taxon because of the edge between metabolite concentration and taxon abundance. To address the apparent disconnect of the time scales between the data set (2 weeks) and the laboratory experiments (several hours), we also subsampled the original data set with a sampling rate of 1 day and learned independent networks, making sure that the tested interactions were also in these densely sampled networks.

After plotting the growth curves with the bacterium and metabolite in question, using a two-tailed paired *t* test, we assessed if each metabolite was enhancing/inhibiting the taxon growth in comparison to growth without the metabolite.

### Preliminary experiments.

Three preliminary trials are outlined below, which paved the way for the final experiments that were run. The bacterial strains used, Escherichia coli HB101 (32) and Pseudomonas aeruginosa PAO1 (31), were routinely cultured in Luria-Bertani (LB 20%) broth (5 g tryptone, 10 g sodium chloride, and 5 g yeast extract per liter) or agar (LB broth with 1.5% agar) (Difco, NJ, USA). Growth curve assays were performed in media supplemented with the metabolites at 37°C. For the three preliminary experiments, we attempted to mimic a limited-nutrient environment by using minimal medium [MM; in grams/liter, (NH_4_)_2_SO_4_, 2.0; K_2_HPO_4_, 0.5; MgSO_4_ · 7H_2_O, 0.2; FeSO_4_ · 7H_2_O, 0.01, pH 7.2 ± 0.2]. E. coli was grown in the presence of 4-methylcatechol (4-MC, C_7_H_8_O_2_) and 4-hydroxyphenylacetate (4-HPA, C_8_H_8_O_3_). P. aeruginosa was grown in the presence of d-xylose (C_5_H_10_O_5_), and 1-methylnicotinamide (1-MNA, C_7_H_9_N_2_O^+^).

In Experiment 1, bacteria were grown in 0.2 M of MM mixed with 0.2 mM glucose and 0.2 mM metabolites. The cells reached the stationary phase at a very low OD, suggesting that this is not the suitable medium to be used. The metabolites did not affect the bacterial growth at the exponential phase.

In Experiment 2, the effect of metabolites on bacterial growth at the stationary phase was tested using MM combined with 20% Luria Bertani broth with metabolites at 0.2, 1.0, and 2 mM. The cells reached the stationary phase at a higher OD. The metabolites at lower concentrations did not affect the exponential phase; however, they are lethal at 2 mM.

In Experiment 3, similar conditions in experiment 2 were repeated (MM plus 20% Luria Bertani broth) but using only 0.2 and 1.0 mM metabolites individually. The metabolite effect on bacterial growth was observed only at the stationary phase.

### Final experiments.

Because no significant difference was observed in the exponential growth rate and consequently in the doubling time under all the conditions tested for both E. coli and P. aeruginosa, we ran a new test in which the metabolites were added at the beginning of the stationary phase to test its effect on it.

The growth of E. coli was monitored hourly in the absence (control) and presence of 4-MC, 4-HPA, d-xylose, and glucose at 0.2 and 1 mM. Glucose and d-xylose were used as enhancer positive controls. At the lower concentration (0.2 mM) in a comparison with the control (LB 20%), 4-HPA had no effect and 4-MC, d-xylose, and glucose were enhancing (Fig. S9a and c). The compound 4-HPA has no effect at a low concentration; however, at 1 mM, there is a significant enhancing effect starting at early stationary phase (Table 1). At the highest concentration, all metabolites produce an enhancer effect that is statistically significant (*t* test, *P* < 0.05); there is also a more pronounced enhancer effect of 4-MC and glucose than of d-xylose and 4-HPA (Fig. 6).

The growth of P. aeruginosa PAO1 was monitored hourly in the absence (control) and presence of d-xylose, 1-MNA, and succinate at 0.2 and 1 mM. Succinate was used as the enhancer positive control, and 1-MNA was used as the negative control. It is worth noting that 1-MNA does not appear in any of our learned networks: alignment, no-alignment, Skeleton, Augmented, or any number of parents tested. d-Xylose and succinate at 0.2 mM appear to have an enhancer effect in the stationary phase (Fig. S9b), but the differences are not statistically significant at this concentration (Fig. S9d). No effect was observed on P. aeruginosa growth in the presence 1-MNA. At a 1 mM concentration, d-xylose and succinate produced and enhanced growth, and the difference was statistically significant (Table 1) (*t* test, *P* < 0.05). The presence of 1-MNA did not have a significant effect on P. aeruginosa growth; it could potentially be an inhibitory compound (Fig. 6).

### Data citation.

All data analyzed in this work are derived from the iHMP IBD website (https://www.ibdmdb.org; 13).

### Data availability.

Source code and data are freely available on our GitHub page (https://github.com/DaniRuizPerez/PALM-Public-Respository), linked from the project website (http://biorg.cs.fiu.edu/palm/), under the MIT Open Source license agreement.
