# Supplementary material for: Dynamic Bayesian Networks for Integrating Multi-omics Time Series Microbiome Data
Source: mSystems. 2021 Mar 30;6(2):e01105-20. doi: 10.1128/mSystems.01105-20 (PMC8546994; doi:10.1128/mSystems.01105-20)
Supplement: FIG S1 [file msystems.01105-20-sf001.pdf]

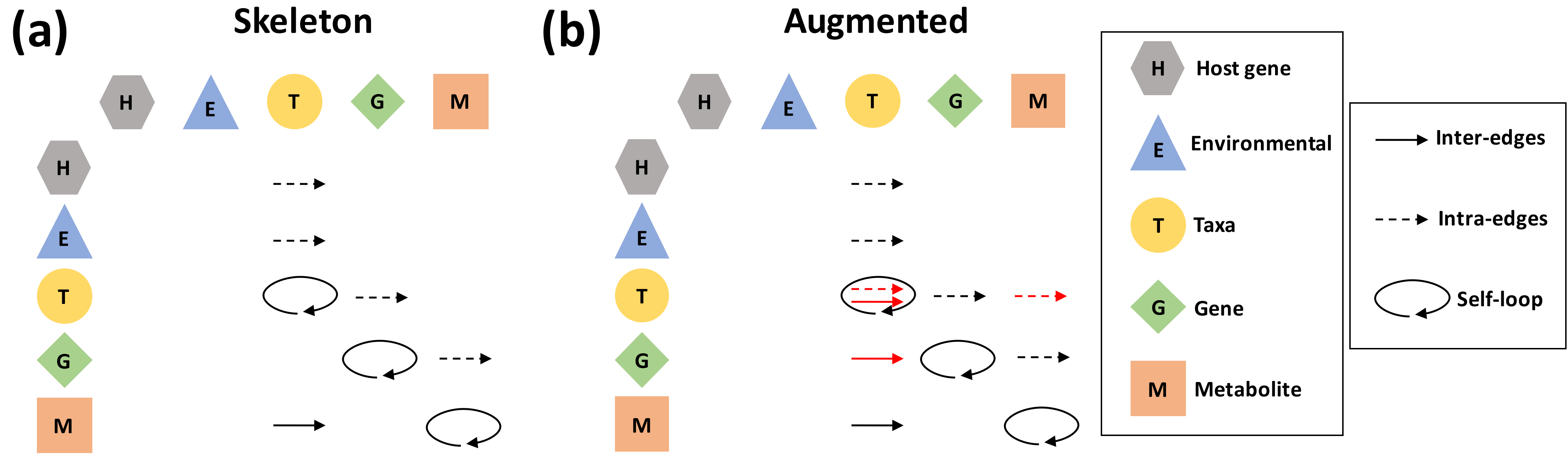

(a)
Skeleton
(b)
Augmented
H
E
T
G
M
Host gene
Environmental
Taxa
Gene
Metabolite
Inter-edges
Intra-edges
Self-loop
