# Supplementary figures and images for: Dynamic Bayesian Networks for Integrating Multi-omics Time Series Microbiome Data
Source: mSystems. 2021 Mar 30;6(2):e01105-20. doi: 10.1128/mSystems.01105-20 (PMC8546994; doi:10.1128/mSystems.01105-20)

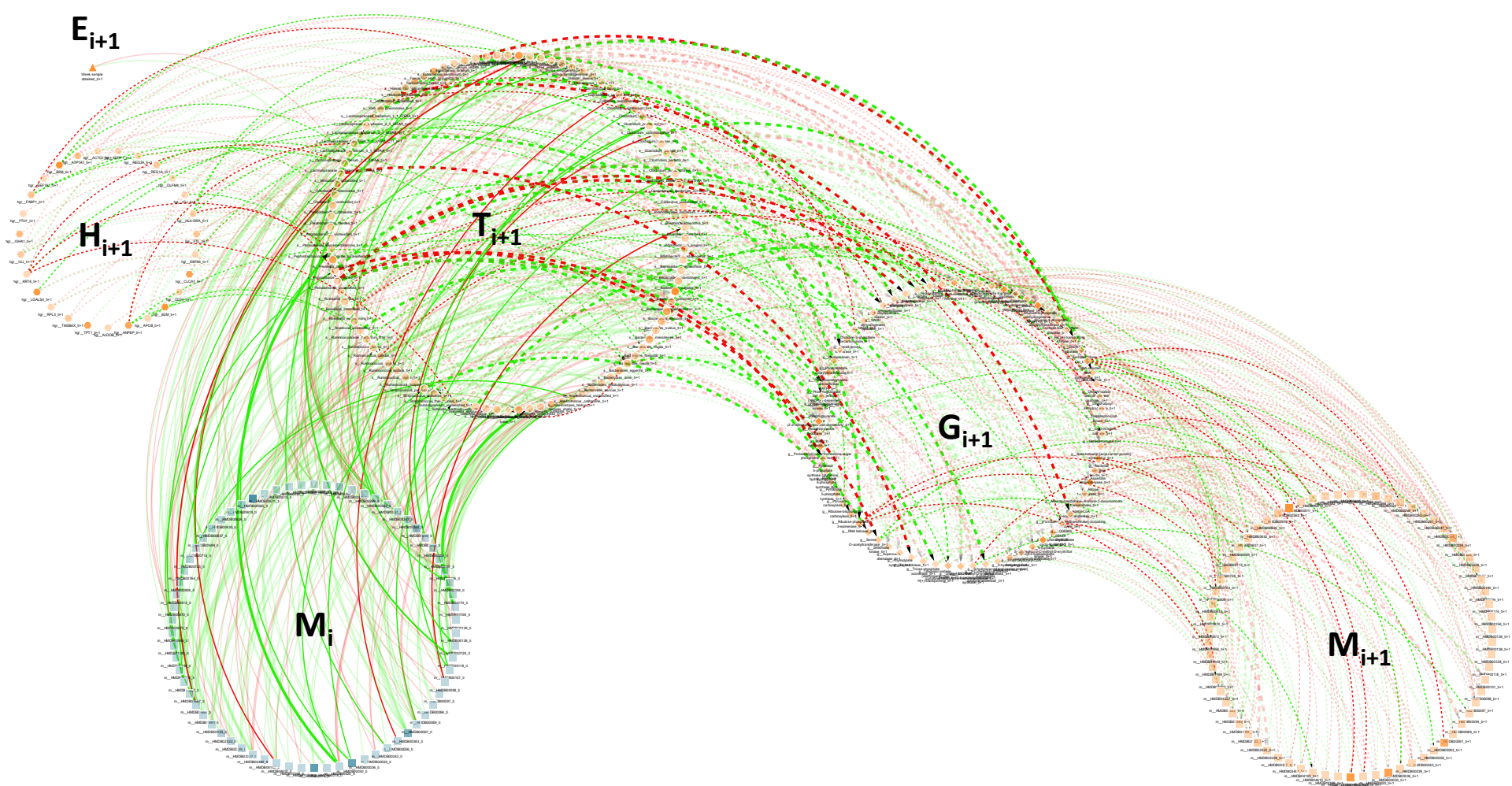

Supplement: FIG S2 [file msystems.01105-20-sf002.pdf]

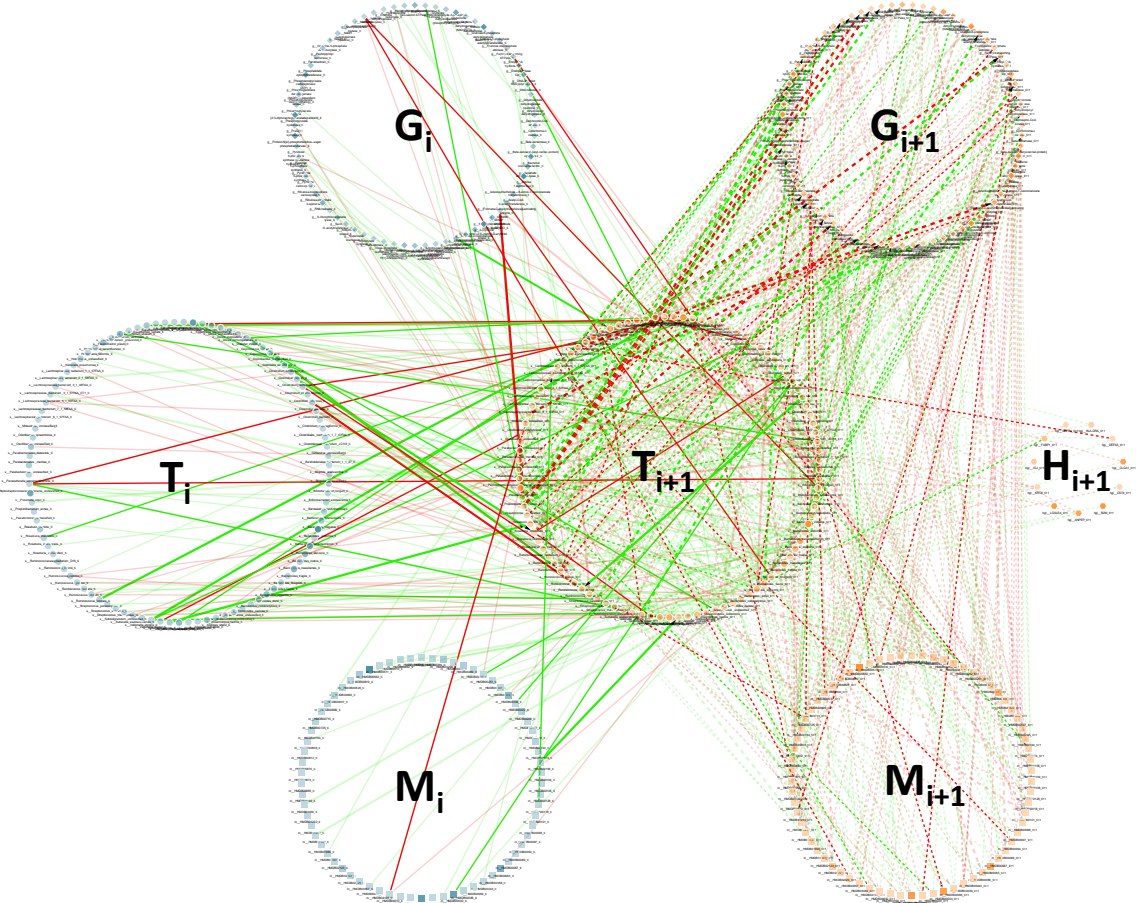

Supplement: FIG S3 [file msystems.01105-20-sf003.pdf]

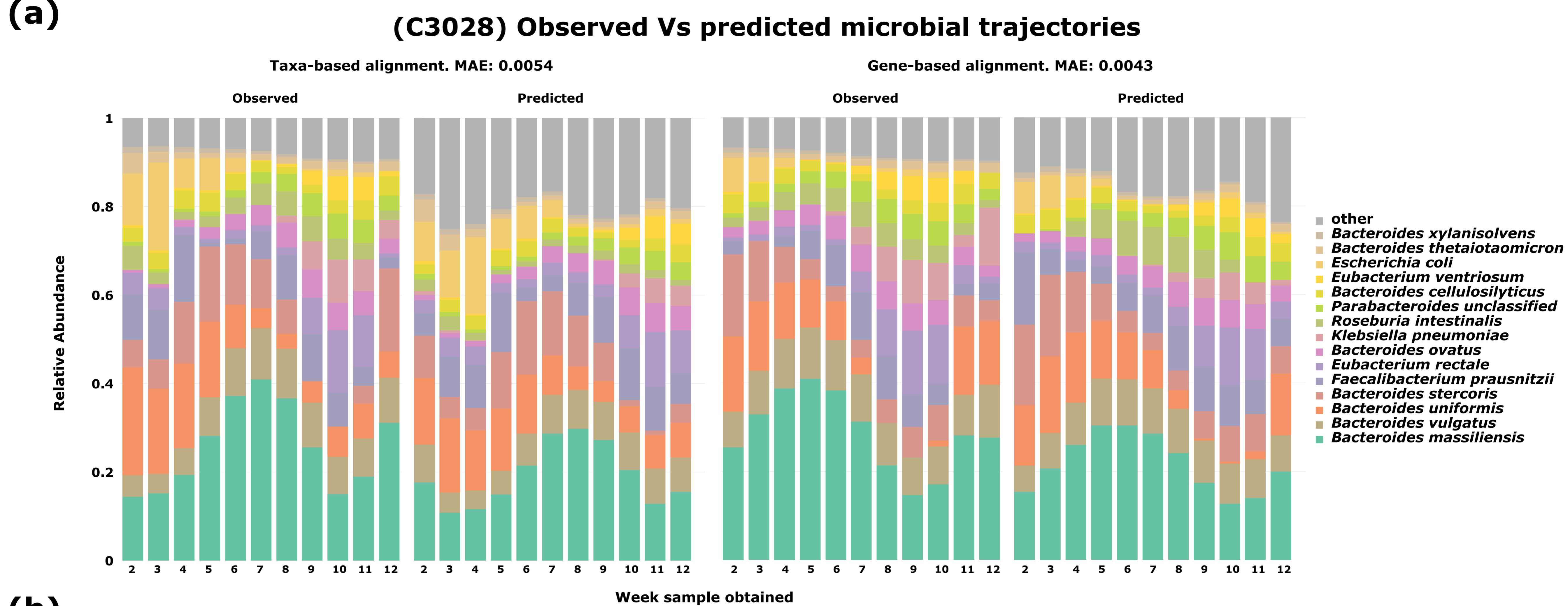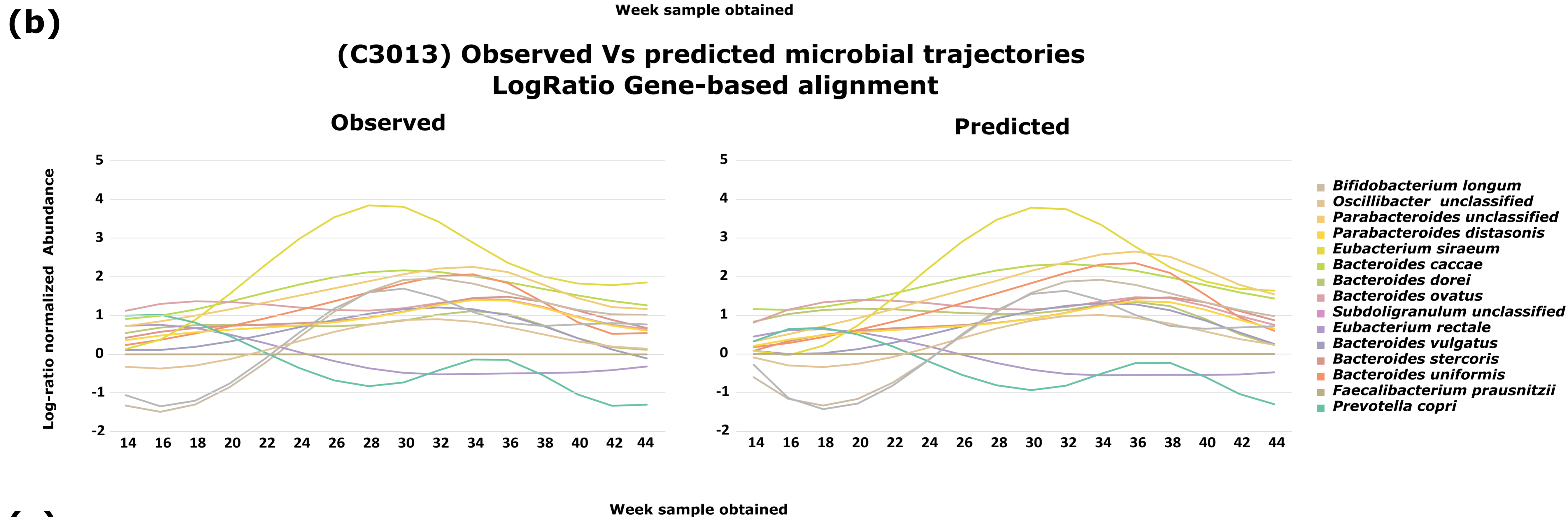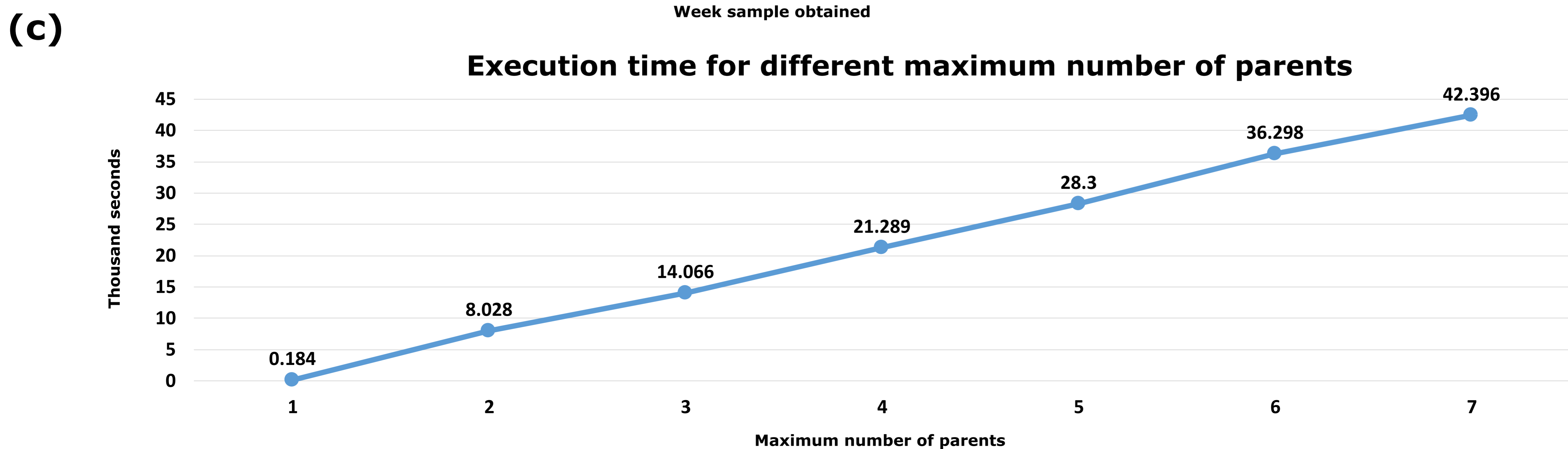

Supplement: FIG S4 [file msystems.01105-20-sf004.pdf]

**(a)**

# MAE of different alignments in PALM

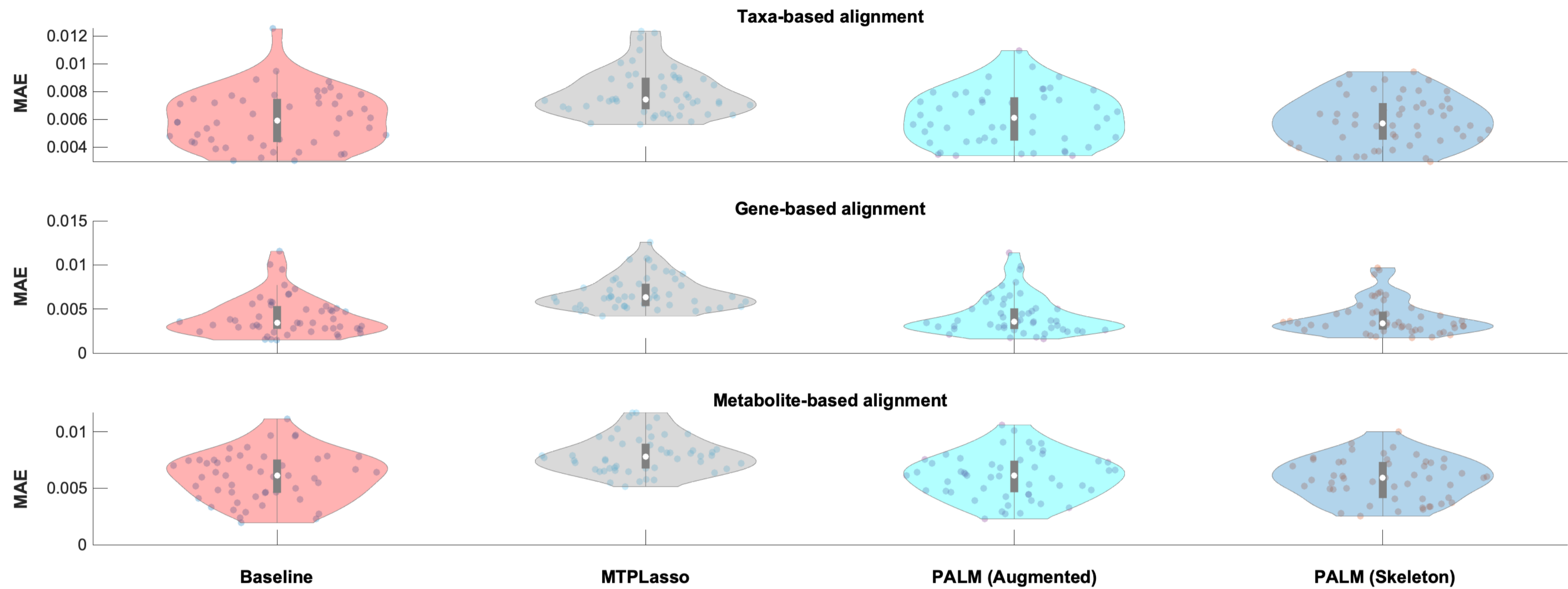**(b)**

# RMSE of PALM Vs MMvec

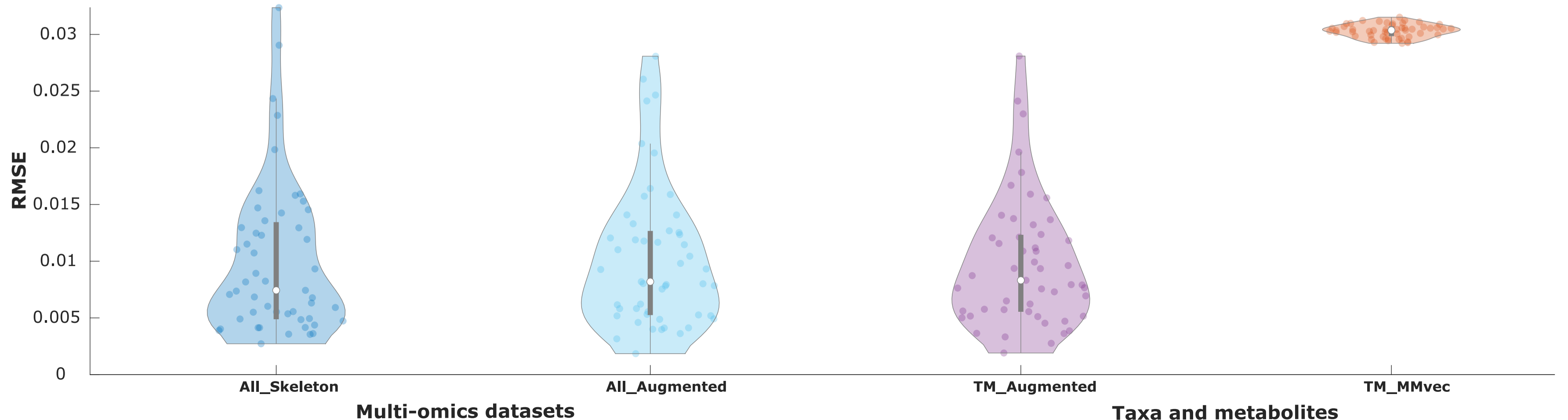

Supplement: FIG S5 [file msystems.01105-20-sf005.pdf]

**(a)**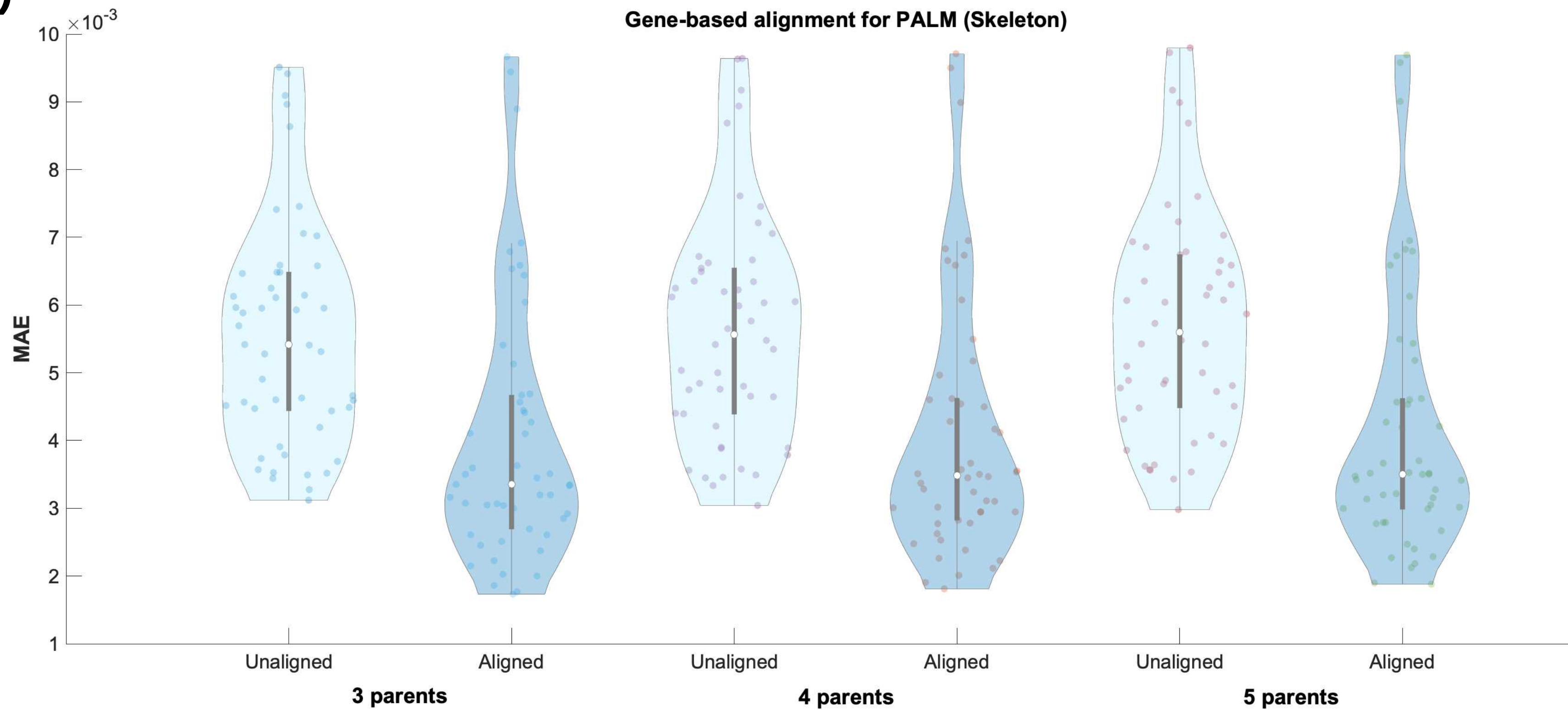**(b)**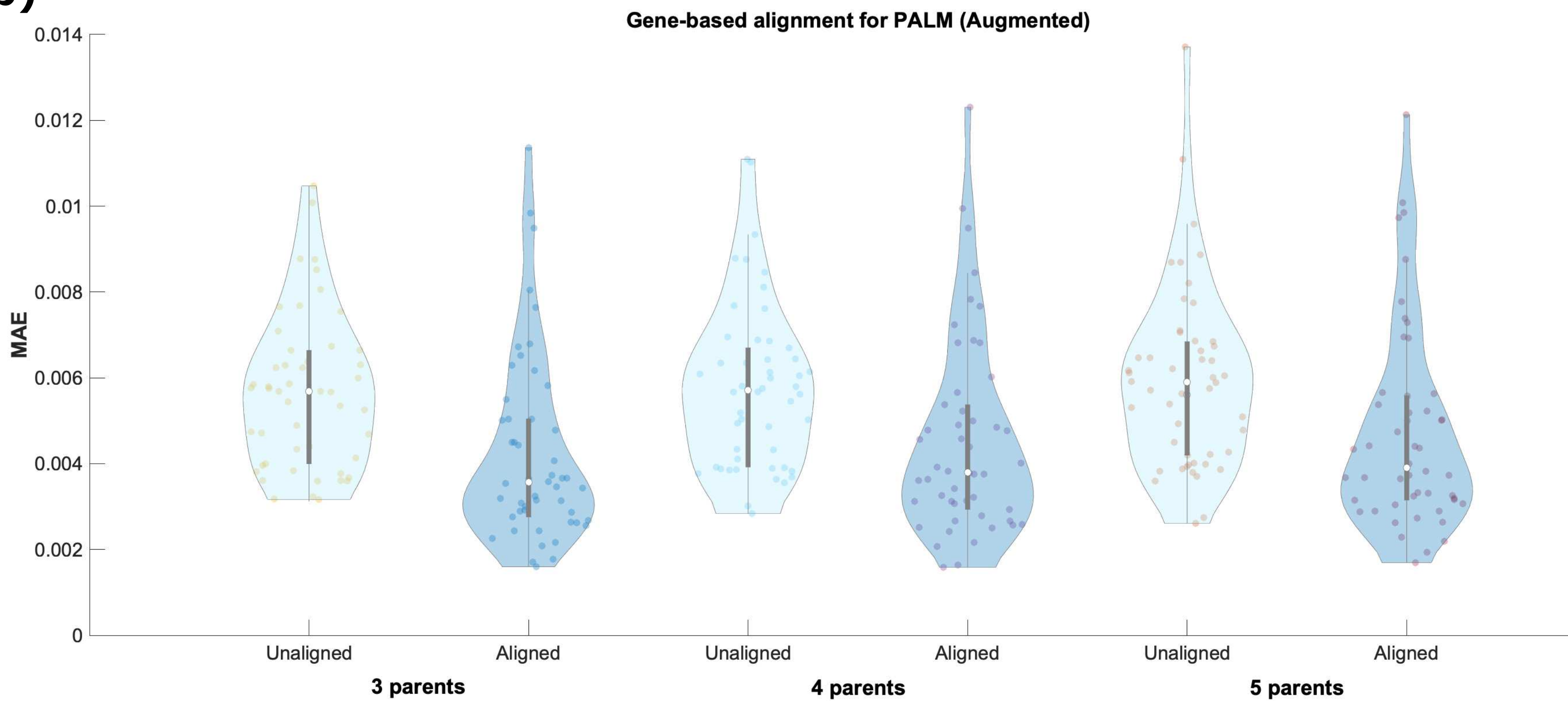

Supplement: FIG S6 [file msystems.01105-20-sf006.pdf]

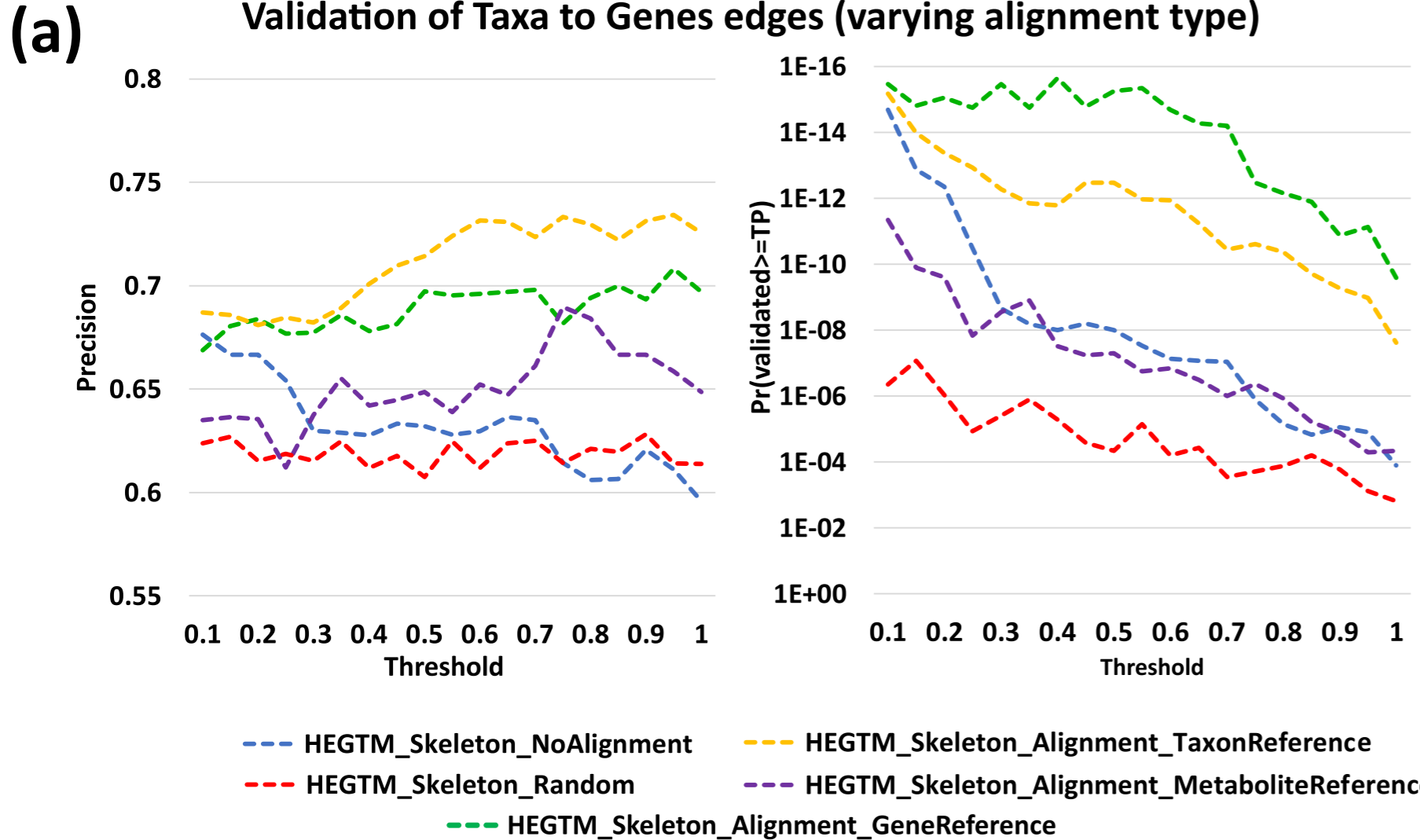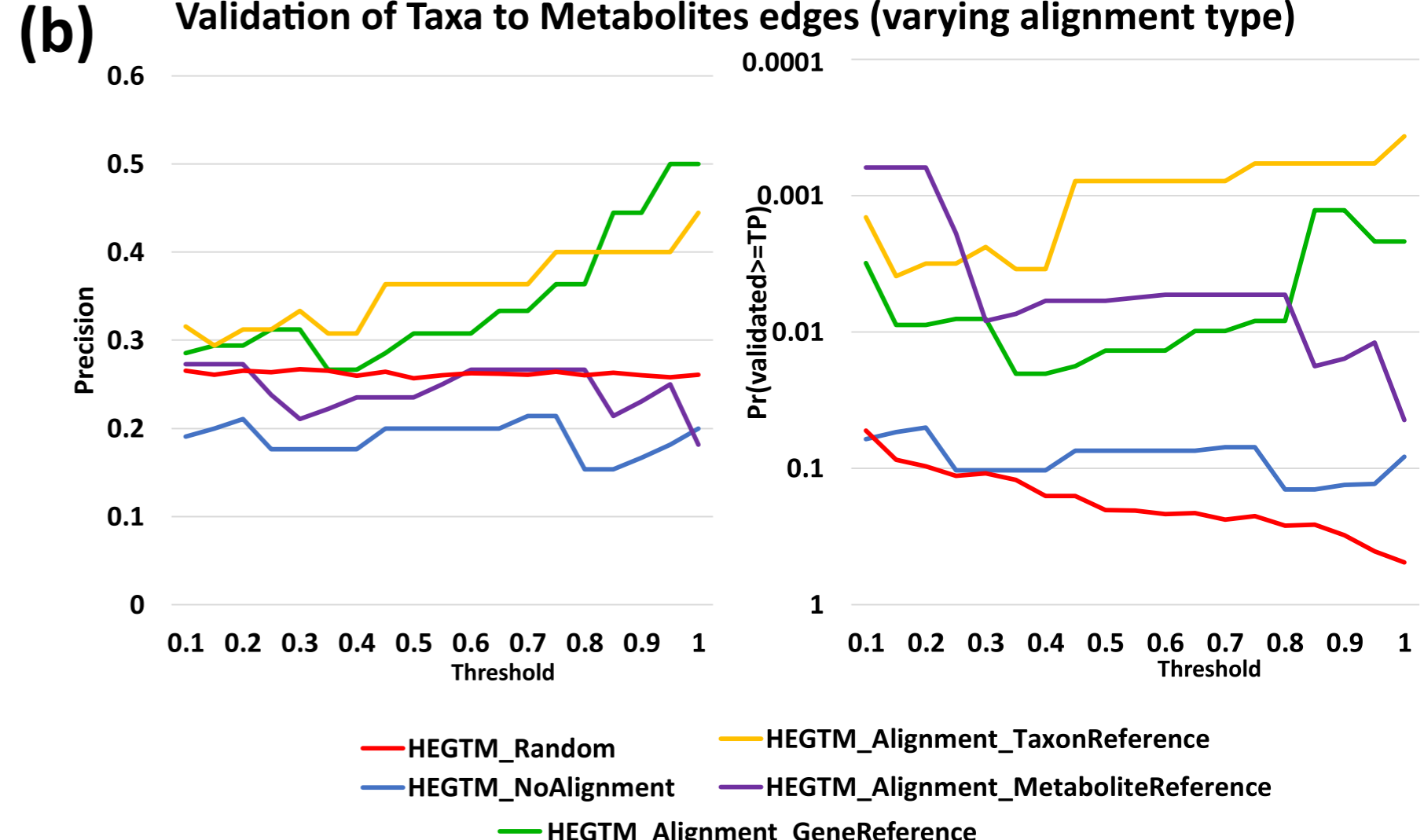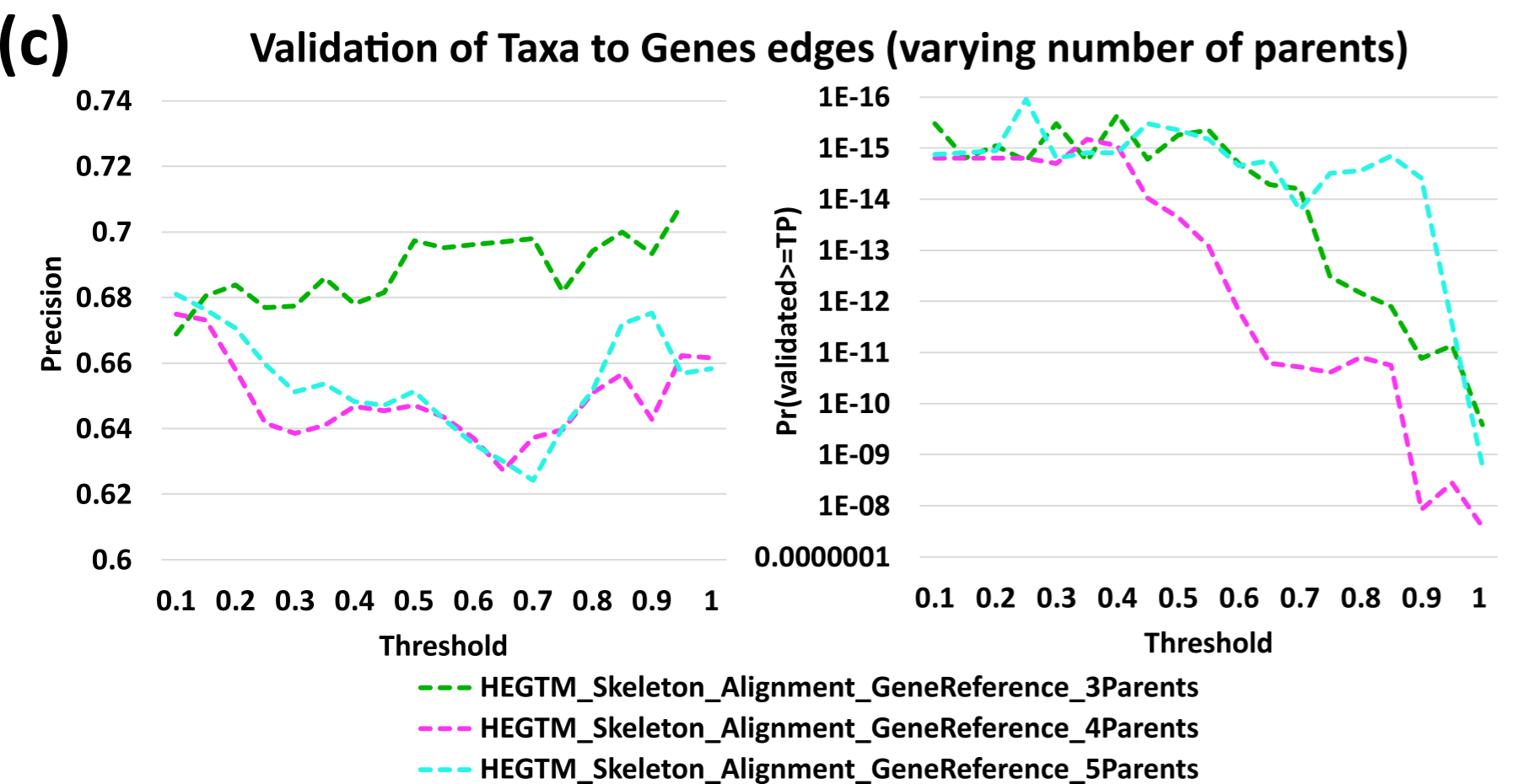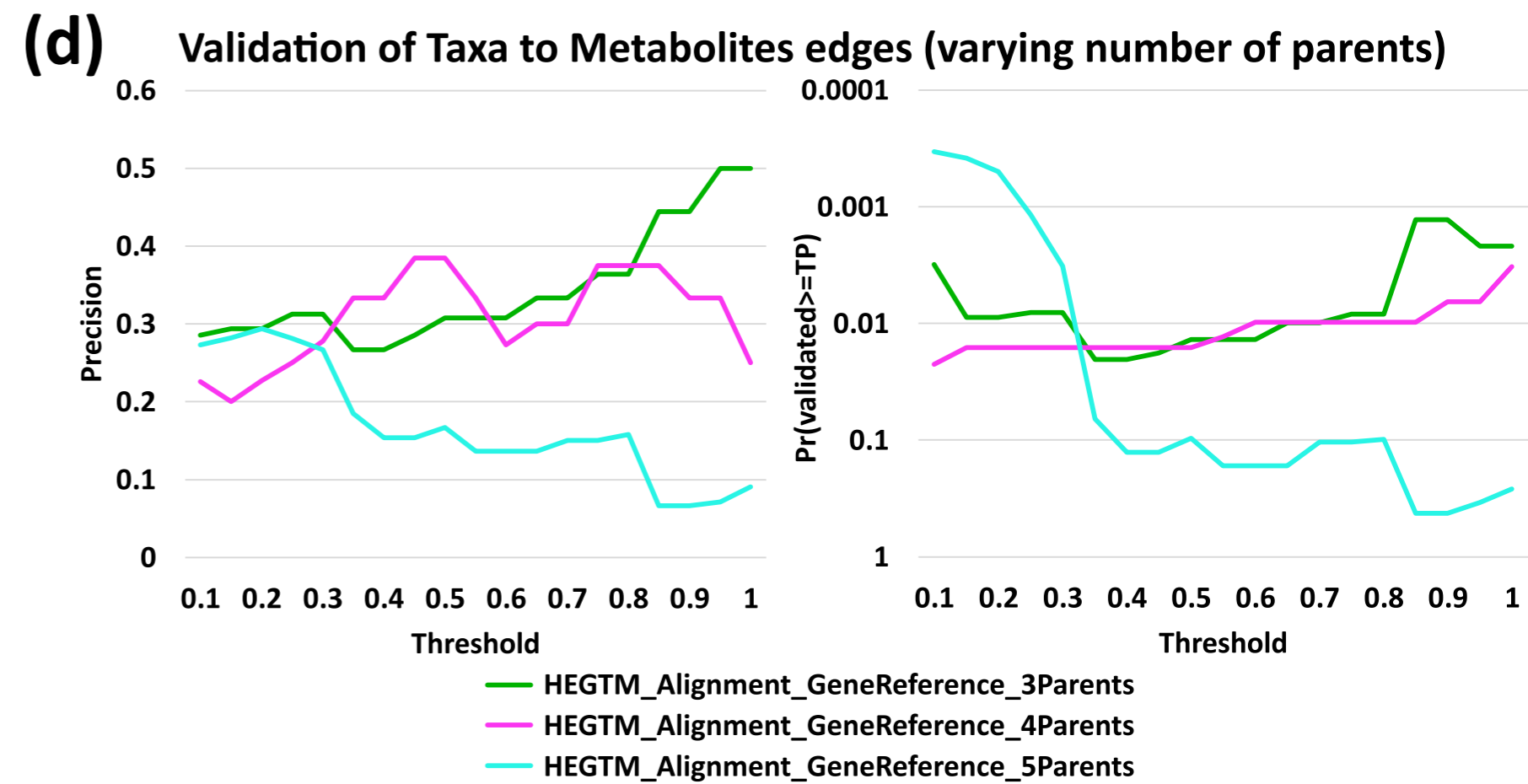

Supplement: FIG S7 [file msystems.01105-20-sf007.pdf]

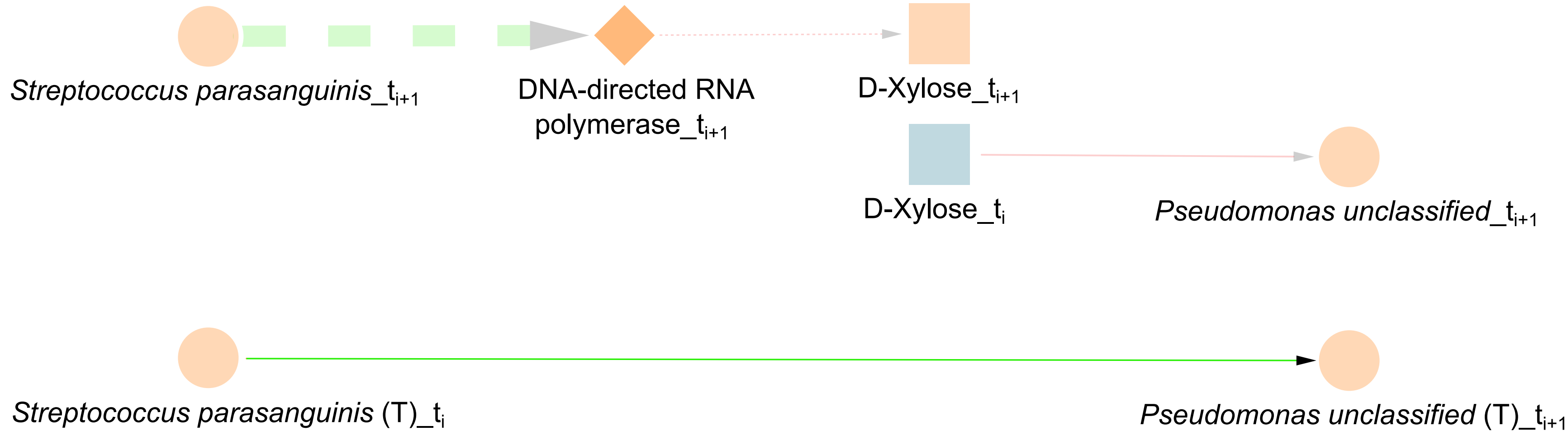

Supplement: FIG S10 [file msystems.01105-20-sf010.pdf]
