# Supplementary material for: Dynamic Bayesian Networks for Integrating Multi-omics Time Series Microbiome Data
Source: mSystems. 2021 Mar 30;6(2):e01105-20. doi: 10.1128/mSystems.01105-20 (PMC8546994; doi:10.1128/mSystems.01105-20)
Supplement: FIG S8 [file msystems.01105-20-sf008.pdf]

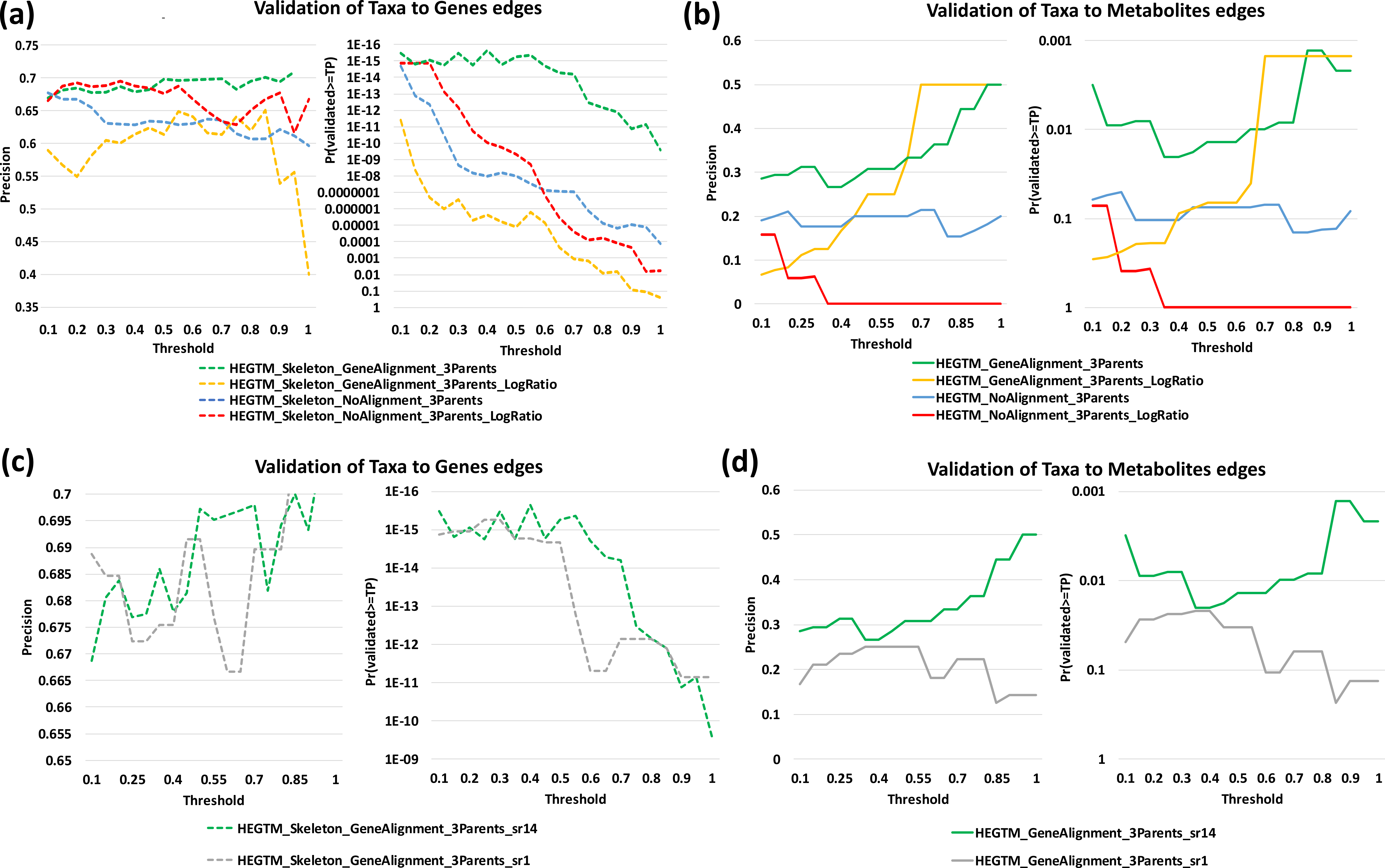

(a)
Validation of Taxa to Genes edges
Precision
Pr(validated>=TP)
Threshold
Threshold
HEGTM_Skeleton_GeneAlignment_3Parents
HEGTM_Skeleton_GeneAlignment_3Parents_LogRatio
HEGTM_Skeleton_NoAlignment_3Parents
HEGTM_Skeleton_NoAlignment_3Parents_LogRatio
(b)
Validation of Taxa to Metabolites edges
Precision
Pr(validated>=TP)
Threshold
Threshold
HEGTM_GeneAlignment_3Parents
HEGTM_GeneAlignment_3Parents_LogRatio
HEGTM_NoAlignment_3Parents
HEGTM_NoAlignment_3Parents_LogRatio
(c)
Validation of Taxa to Genes edges
Precision
Pr(validated>=TP)
Threshold
Threshold
HEGTM_Skeleton_GeneAlignment_3Parents_sr14
HEGTM_Skeleton_GeneAlignment_3Parents_sr1
(d)
Validation of Taxa to Metabolites edges
Precision
Pr(validated>=TP)
Threshold
Threshold
HEGTM_GeneAlignment_3Parents_sr14
HEGTM_GeneAlignment_3Parents_sr1
