# Supplementary material for: Dynamic Bayesian Networks for Integrating Multi-omics Time Series Microbiome Data
Source: mSystems. 2021 Mar 30;6(2):e01105-20. doi: 10.1128/mSystems.01105-20 (PMC8546994; doi:10.1128/mSystems.01105-20)
Supplement: FIG S9 [file msystems.01105-20-sf009.pdf]

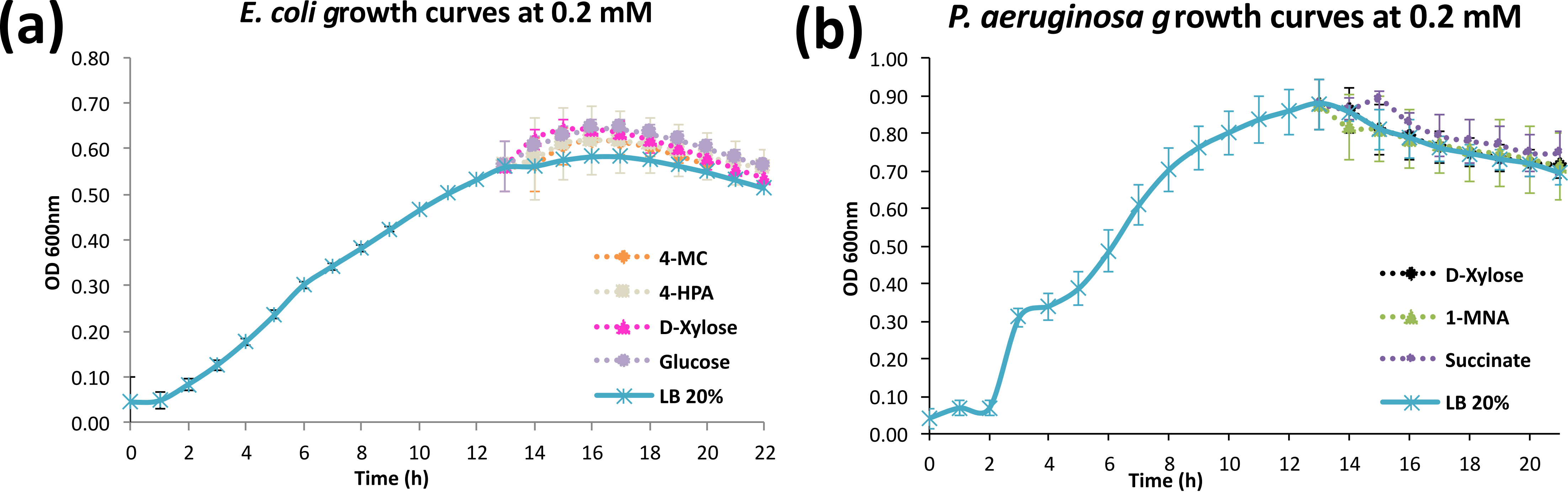

(c)

Escherichia coli HB101			
**Met.**	**16h**	**17h**	**18h**
LB	0.583	0.584	0.576
4-MC	0.617 (0.0108)	0.614 (0.0055)	0.602 (0.0026)
4-HPA	0.617 (0.1458)	0.616 (0.1332)	0.608 (0.1049)
D-Xylose	0.642 (0.0028)	0.635 (0.0073)	0.619 (0.0132)
Glucose	0.644 (0.0032)	0.644 (0.0036)	0.633 (0.0049)

(d)

Pseudomonas aeruginosa PAO1			
**Met.**	**15h**	**16h**	**17h**
LB	0.811	0.787	0.760
D-Xylose	0.812 (0.9029)	0.793 (0.7561)	0.763 (0.7710)
1-MNA	0.813 (0.9117)	0.786 (0.9416)	0.772 (0.6110)
Succinate	0.889 (0.0128)	0.830 (0.6979)	0.795 (0.0530)
